# Bcl-3 promotes Wnt signaling by maintaining the acetylation of β-catenin at lysine 49 in colorectal cancer

**DOI:** 10.1038/s41392-020-0138-6

**Published:** 2020-05-01

**Authors:** Xi Chen, Chen Wang, Yuhang Jiang, Qi Wang, Yu Tao, Haohao Zhang, Yongxu Zhao, Yiming Hu, Cuifeng Li, Deji Ye, Dandan Liu, Wenxia Jiang, Eugene Y. Chin, Sheng Chen, Yongzhong Liu, Mingliang Wang, Sanhong Liu, Xiaoren Zhang

**Affiliations:** 1 CAS Key Laboratory of Tissue Microenvironment and Tumor, Shanghai Institute of Nutrition and Health, Shanghai Institutes for Biological Sciences, University of Chinese Academy of Sciences, Chinese Academy of Sciences, Shanghai, 200025 China; 2 Shanghai Institute for Advanced Immunochemical Studies, ShanghaiTech University, Shanghai, 201210 China; 3 Affiliated Cancer Hospital & Institute of Guangzhou Medical University, Guangzhou Municipal and Guangdong Provincial Key Laboratory of Protein Modification and Degradation, State Key Laboratory of Respiratory Disease, Guangzhou, 510000 China; 4 Renji Hospital, Shanghai Jiao Tong University School of Medicine, Shanghai, 200025 China; 5 State Key Laboratory of Oncogenes and Related Genes, Shanghai Cancer Institute, Renji Hospital, Shanghai Jiao Tong University School of Medicine, Shanghai, 200032 China; 6 Department of General Surgery, Ruijin Hospital, Shanghai Jiao Tong University School of Medicine, Shanghai, 200025 China

**Keywords:** Cancer stem cells, Gastrointestinal cancer

## Abstract

Wnt/β-catenin signaling plays a critical role in colorectal cancer (CRC) tumorigenesis and the homeostasis of colorectal cancer stem cells (CSCs), but its molecular mechanism remains unclear. B-cell lymphoma 3 (Bcl-3), a member of the IκB family, is overexpressed in CRC and promotes tumorigenicity. Here, we report a novel function of Bcl-3 in maintaining colorectal CSC homeostasis by activating Wnt/β-catenin signaling. Silencing Bcl-3 suppresses the self-renewal capacity of colorectal CSCs and sensitizes CRC cells to chemotherapeutic drugs through a decrease in Wnt/β-catenin signaling. Moreover, our data show that Bcl-3 is a crucial component of Wnt/β-catenin signaling and is essential for β-catenin transcriptional activity in CRC cells. Interestingly, Wnt3a increases the level and nuclear translocation of Bcl-3, which binds directly to β-catenin and enhances the acetylation of β-catenin at lysine 49 (Ac-K49-β-catenin) and transcriptional activity. Bcl-3 depletion decreases the Ac-K49-β-catenin level by increasing the level of histone deacetylase 1 to remove acetyl groups from β-catenin, thus interrupting Wnt/β-catenin activity. In CRC clinical specimens, Bcl-3 expression negatively correlates with the overall survival of CRC patients. A significantly positive correlation was found between the expression of Bcl-3 and Ac-K49-β-catenin. Collectively, our data reveal that Bcl-3 plays a crucial role in CRC chemoresistance and colorectal CSC maintenance via its modulation of the Ac-K49-β-catenin, which serves as a promising therapeutic target for CRC.

## Introduction

Colorectal cancer (CRC) is the fourth leading cause of cancer deaths, and the incidence rate ranks third worldwide.^1^ Cancer stem cells (CSCs) have the capacity for self-renewal, tumor initiation, tumor growth, metastasis,^2^ and resistance to chemotherapy.^3^ Therefore, the elucidation of the mechanisms that maintain colorectal CSCs is essential for the development of new therapeutic treatments in CRC.

Colorectal CSCs can be identified by a variety of cell surface markers, including CD133, CD44, and CD24.^4,5^ Persistent activation of canonical Wnt signaling is crucial for the tumorigenesis of CRC (refs. ^6,7^) and stemness maintenance of colorectal CSCs.^8,9^ Wnt signaling is activated when Wnt ligands bind to the membrane receptors Frizzled and LDL receptor-related protein 5 or 6. Subsequently, β-catenin in the cytoplasm is released from the β-catenin destruction complex, and stabilized β-catenin translocates to the nucleus to form a transcriptional complex with LEF/TCF.^6^ In the absence of Wnt ligand treatment, β-catenin in the cytoplasm is rapidly degraded by a multiprotein complex that includes APC (*adenomatous polyposis coli*), AXIN, and GSK-3 (glycogen synthase kinase-3) kinase. One of the core components of the Wnt/β-catenin destruction complex is GSK-3. Under normal circumstances, GSK-3 phosphorylates β-catenin on its N-terminal Thr41, Ser37, and Ser33 residues.^10^ Thereafter, phosphorylated β-catenin is ubiquitinated and degraded by the proteasome.^11^

B-cell lymphoma 3 (Bcl-3) is a member of the IκB family, and it is an important transcriptional regulator that forms complexes with p50 and p52 on DNA.^12,13^ Bcl-3 promotes breast cancer metastasis^14^ by stabilizing Smad3/TGFβ signaling.^15^ In glioblastoma cells, Bcl-3 significantly facilitated proliferation by stabilizing the expression of STAT3, p-STAT3, and STAT3 signaling target genes, including Bcl-2, MCL-1, and cyclin D1.^16^ Recently, the function of Bcl-3 in CRC has been reported by various research groups. First, Bcl-3 was found to promote CRC cell growth by stabilizing the c-Myc protein level and regulating ERK signaling.^17^ In addition, Bcl-3 promotes CRC tumorigenesis based on the activation of AKT signaling.^18^ Finally, Bcl-3 suppresses inflammation-associated colon tumorigenesis in epithelial cells by dampening tumorigenic NF-κB signaling.^19^ Moreover, previously uncovered evidence demonstrated the function of Bcl-3 in pluripotency maintenance. Bcl-3 functions as a downstream molecule of LIF/STAT3 signaling and positively regulates pluripotency genes, including *Oct4*, *Sox2*, and *Nanog*, in mouse embryonic stem cells (mESCs).^20,21^

Due to the overactivation of Bcl-3 and β-catenin that occurs in many cancers, we hypothesize that there is a potential functional relationship between these two transcriptional regulators. In this study, we explored the possibility of Bcl-3 as a component in Wnt/β-catenin signaling and determined the biological role of Bcl-3 in CRC progression, as well as in colorectal CSCs.

## Results

### Bcl-3 promotes a stem cell-like phenotype in CRC cells in vitro

**Fig. 1:**
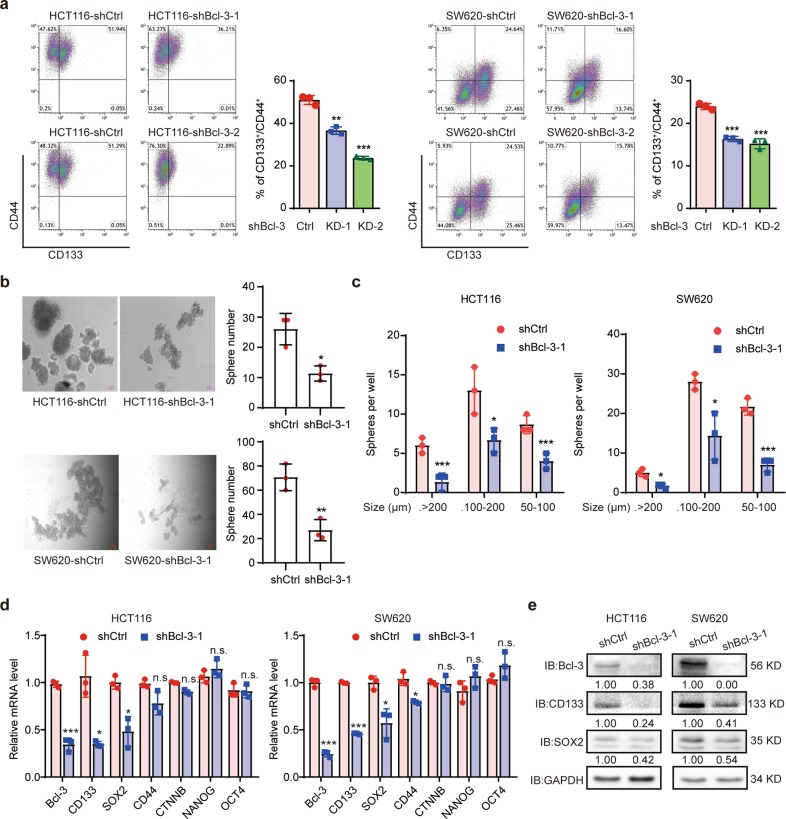
Bcl-3 maintains stem cell-like properties in CRC cells in vitro. **a** CD133^+^CD44^+^ subpopulations were detected in Bcl-3-depleted HCT116 and SW620 cells by FACS analyses, and plotted as the percentage of positive cells. The results are shown as the means ± SD. *p < 0.05, **p < 0.01, and ***p < 0.001 by two-tailed Student’s *t*-test. **b** Bcl-3 depletion causes a reduced sphere-forming capacity in the CRC cell lines HCT116 and SW620. The left panel shows representative images, and the right panel represents the number of spheres formed by the indicated cells. *p < 0.05, **p < 0.01, and ***p < 0.001 by two-tailed Student’s *t*-test. **c** The size of spheres derived from the indicated cells in **b**. *p < 0.05, **p < 0.01, and ***p < 0.001 by two-tailed Student’s *t*-test. **d** q-RT-PCR analysis of the expression level of CSC-related genes (*CD133*, *SOX2*, *CD44*, *CTNNB1*, *NANOG*, and *OCT4*) and *Bcl-3* in Bcl-3-silenced cells compared with control cells. The results are expressed as the means ± SD for each cohort (*n* = 3). *p < 0.05, **p < 0.01, and ***p < 0.001 by two-tailed Student’s *t*-test. **e** Immunoblots for Bcl-3, CD133, and SOX2 in HCT116 and SW620 cells

Prompted by the results of previous studies in which Bcl-3 has a potential oncogenic role in CRC,^17^ as well as the research that Bcl-3 is necessary for pluripotency and self-renewal of mESCs,^20,21^ we hypothesized that Bcl-3 may play an important role in maintaining the homeostasis of colorectal CSCs. The in vitro stemness capability of cells was assessed by flow cytometry to examine the percentage of cells coexpressing CD44 and CD133.^22–24^ The knockdown (KD) efficiency of two different shRNA sequences targeting Bcl-3 is shown in Supplementary Fig. 1a. Bcl-3 KD remarkably impaired the generation of CD44^+^CD133^+^ cells in CRC cells (Fig. 1a). Additionally, Bcl-3 depletion dramatically reduced the number and average diameter of spheres in tumorsphere formation assays in HCT116 and SW620 cells (Fig. 1b, c). Therefore, these results indicate that Bcl-3 is required for the homeostasis of colorectal CSCs and that it promotes the proliferation of CSCs.

Then, we detected the expression of several CSC-related genes, such as *CD44*, *CD133*, *SOX2, OCT4, NANOG*, and *CTNNB1*. The results of q-RT-PCR revealed that the expression of *CD133* and *SOX2* was significantly reduced in Bcl-3 KD cells. The mRNA level of *CTNNB1* barely changed in both cell lines when Bcl-3 was silenced (Fig. 1d). Then, we performed immunoblot assays to confirm the downregulation of SOX2 and CD133 in both cell lines (Fig. 1e). To further assess the relevance between Bcl-3 and CSC-related genes, we analyzed the expression of *Bcl-3*, *SOX2*, and *CD133* in 148 patient samples from the bioinformatics website R2: Genomics Analysis and Visualization Platform (http://r2.amc.nl). Linear regression analyses showed that the mRNA level of Bcl-3 was positively correlated with *CD133* and *SOX2* (Supplementary Fig. 1b). Together, these results indicate that Bcl-3 maintains the stemness of CRCs by regulating the expression of stemness-related genes.

### Bcl-3 enhances tumorigenicity, and Bcl-3 depletion enhances drug sensitivity

**Fig. 2:**
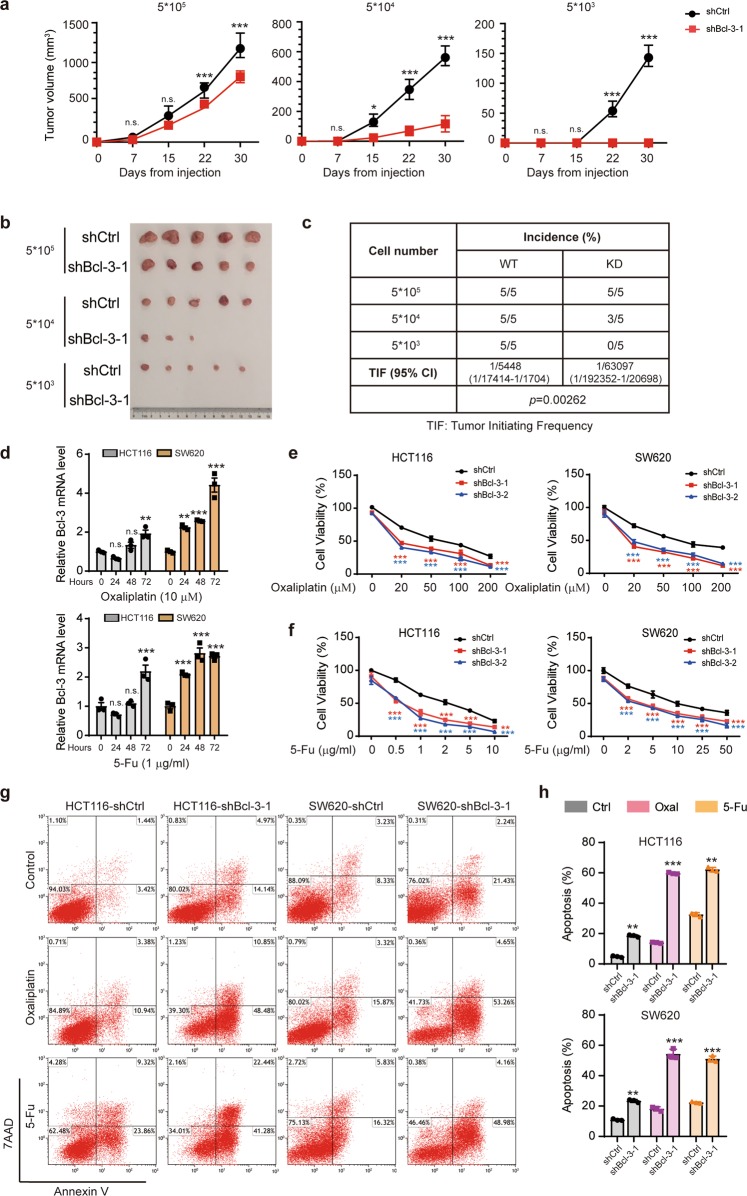
Bcl-3 enhances the tumorigenicity and chemoresistance of CRC in vivo. **a** A total of 5 × 10^5^, 5 × 10^4^, and 5 × 10^3^ Bcl-3-silenced (shBcl-3-KD-1) HCT116 cells or control cells were subcutaneously inoculated into BALB/c nude mice for observation of tumor growth. The results are shown as the means ± SD; (*n* = 5 for each group). *Adjusted *p* value < 0.05; **adjusted *p* value < 0.01; and ***adjusted *p* value < 0.001 by two-way ANOVA. **b** Representative images of tumors in **a**, 30 days after injection. **c** Tumorigenic cell frequency in Bcl-3-silenced HCT116 cells or control cells was determined by limiting dilution assays (http://bioinf.wehi.edu.au/software/elda/). **d** q-RT-PCR analysis of *Bcl-3* mRNA expression levels in HCT116 and SW620 cells treated with 5-FU and oxaliplatin for the indicated time points. *Adjusted *p* value < 0.05; **adjusted *p* value < 0.01; and ***adjusted *p* value < 0.001 by one-way ANOVA. **e**, **f** Bcl-3-KD and corresponding control cells were treated with different concentrations of 5-FU or oxaliplatin for 48 h. Cell viability was determined by MTT assay. *Adjusted *p* value < 0.05; **adjusted *p* value < 0.01; and ***adjusted *p* value < 0.001 by two-way ANOVA. **g**, **h** Bcl-3-silenced cells and control cells were treated with 5-FU (1 μg/ml) or oxaliplatin (20 μM) for 48 h as indicated. The percentage of apoptotic cells was determined by flow cytometry

To evaluate the effect of Bcl-3 on the tumorigenicity of CRC cells in vivo, we first confirmed the KD efficiency in HCT116 cells (Supplementary Fig. 2a, b). The shBcl-3-1 sequence was used in the experiments below. Three doses of Bcl-3-silenced HCT116 cells and the corresponding control cells were subcutaneously inoculated into BALB/c nude mice. As shown in Fig. 2a, b, Bcl-3 depletion significantly suppressed xenograft tumor growth and tumorigenic cell frequency. Moreover, Bcl-3 KD led to a >90% reduction in CSC frequency, as demonstrated by in vivo limited dilution assays (Fig. 2c), suggesting that Bcl-3 KD reduced tumor-initiating capacity.

Due to the potential contribution of CSCs in chemoresistance, we wanted to determine whether Bcl-3 is involved in drug resistance. We first assessed the expression of Bcl-3 after 5-fluorouracil (5-FU) and oxaliplatin (Oxal) treatment of HCT116 and SW620 cells. There was a significant increase in the mRNA level of *Bcl-3* after 5-FU or Oxal treatment in both cell lines (Fig. 2d). The same result was found on the online database ONCOMINE Colorectal Dataset (https://www.oncomine.org/resource/main.html)^25^ (Supplementary Fig. 2c). Therefore, we determined the sensitivity of HCT116 and SW620 cells to 5-FU and Oxal after the depletion of Bcl-3, using MTT and FACS assays. Bcl-3 depletion markedly reduced chemoresistance and increased the percentage of apoptotic cells upon treatment with 5-FU and Oxal (Fig. 2e–h). These data suggest that Bcl-3 depletion increases 5-FU- and Oxal-induced cell apoptosis, and enhances drug sensitivity in CRC cells.

### Wnt3a increases Bcl-3 protein expression via GSK-3 kinase activity

**Fig. 3:**
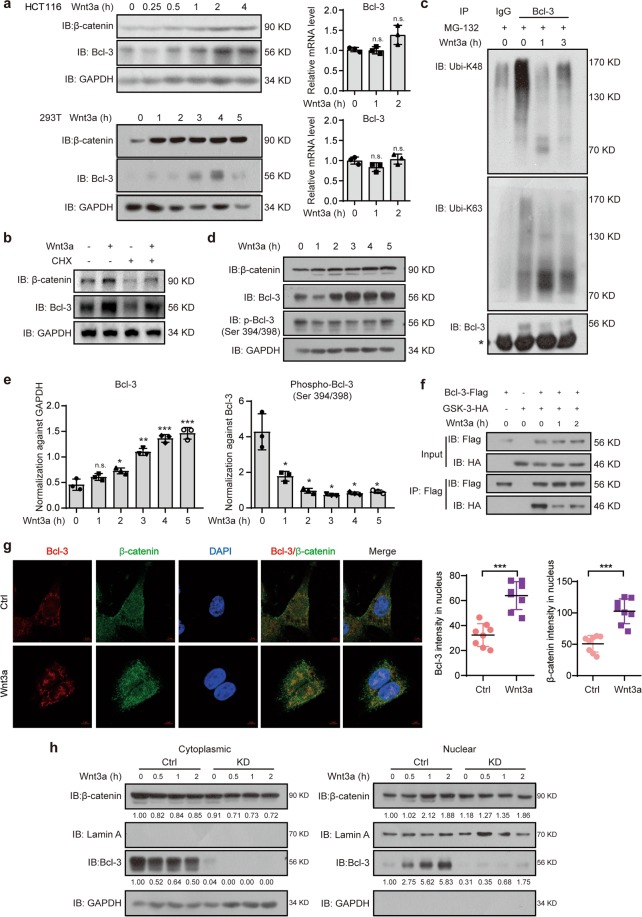
The Wnt3a-induced increase in Bcl-3 protein relies on GSK-3 kinase activity. **a** Immunoblot for Bcl-3 and β-catenin in HCT116 and 293 T cells treated with 100 ng/ml Wnt3a for the indicated times (left). q-RT-PCR analysis of *Bcl-3* mRNA expression levels in HCT116 and 293 T cells treated with 100 ng/ml Wnt3a for the indicated time points (right). **b** HCT116 cells were treated with cycloheximide (CHX, 50 mg/ml) for 12 h and 100 ng/ml Wnt3a for 4 h. Then, total cell lysates were analyzed by immunoblots for Bcl-3 and β-catenin. **c** Immunoblot analyses of immunoprecipitated Bcl-3 for the presence of K48- and K63-linked ubiquitin after MG-132 and Wnt3a stimulation. * Represents the heavy chain band. **d** Immunoblot analyses of Bcl-3, p-Bcl-3 (Ser394/398), and β-catenin after Wnt3a stimulation for the indicated time points. **e** The normalized Bcl-3 and p-Bcl-3 (Ser394/398) protein levels detected in **d. f** Co-IP analyses of Flag-tagged Bcl-3 (Bcl-3-Flag) and HA-tagged GSK-3 (GSK-3-HA) expressed in 293 T cells. After treatment with Wnt3a for the indicated times, cell lysates were subjected to IP with anti-Flag antibody. **g** Immunofluorescence staining of Bcl-3 (red) and β-catenin (green) in HCT116 cells with or without Wnt3a stimulation for 2 h. The nuclei were stained using DAPI (blue). ImageJ software was used to quantify the results. **h** Cytoplasmic and nuclear levels of Bcl-3 and β-catenin in HCT116 cells treated with 100 ng/ml Wnt3a for the indicated times were analyzed by immunoblot. The results were quantified by ImageJ software. The relative protein levels of β-catenin and Bcl-3 in the cytoplasm were normalized to GAPDH levels, and the relative protein levels of these two proteins in the nucleus were normalized to Lamin A levels

Bcl-3 can be unregulated by several cytokines,^26–33^ which prompted us to examine whether Bcl-3 responds to Wnt ligands. The expression of Bcl-3 increased after 1 h of Wnt3a treatment and reached maximal induction after 4 h (Fig. 3a). However, *Bcl-3* mRNA levels did not increase upon Wnt3a treatment (Fig. 3a). After inhibition of new protein synthesis by cycloheximide (CHX), Wnt3a treatment resulted in increased Bcl-3 compared with the control treatment, which indicates a decrease in endogenous Bcl-3 degradation after Wnt3a treatment (Fig. 3b). Cells were treated with the proteasome inhibitor MG-132 had an elevated Bcl-3 level (Supplementary Fig. 3a, b). However, Wnt3a stimulation did not increase Bcl-3 expression when the cells were pretreated with MG-132 (Supplementary Fig. 3a, b). These results show that Wnt3a-induced Bcl-3 expression through the ubiquitin-proteasome degradation system. Then, we determined the possible changes in the ubiquitination of Bcl-3. Consistent with the stability data, dramatically lower K48-linked ubiquitination of Bcl-3 was observed in cells treated with Wnt3a than in control cells (Fig. 3c). As GSK-3 binds to and phosphorylates Bcl-3 at Ser394/398, which causes the degradation of Bcl-3 by the ubiquitin-proteasome system,^34^ we confirmed that the GSK-3 inhibitor SB216763 significantly increased Bcl-3 expression (Supplementary Fig. 3c). We then tested whether Wnt3a regulates the interaction and phosphorylation between GSK-3 and Bcl-3. The immunoblot assay showed that Bcl-3 protein increased upon Wnt3a stimulation, while the Ser394/398 phosphorylation of Bcl-3 decreased gradually (Fig. 3d, e). The immunoblot assay revealed that Bcl-3 binds with GSK-3, and the interaction was dramatically decreased when the cells were treated with Wnt3a (Fig. 3f). These results thus indicate that Wnt3a stimulation interrupts the interaction between Bcl-3 and GSK-3, inhibits the phosphorylation of Bcl-3, and then shields Bcl-3 from the ubiquitin-dependent proteasome degradation pathway.

As Wnt3a is known to stabilize β-catenin and increase its nuclear accumulation, we wanted to investigate and determine whether Bcl-3 can be transported to the nucleus upon Wnt activation, similar to β-catenin. Indeed, Bcl-3 and β-catenin were localized in both the cytoplasm and nucleus, and Wnt3a promoted the nuclear translocation of both proteins as determined by immunofluorescence (IF) analysis (Fig. 3g). As shown in Fig. 3h, Wnt3a significantly promoted the nuclear translocation of endogenous Bcl-3, as indicated by immunoblot analysis. In addition, neither the expression nor nuclear translocation of β-catenin changed when Bcl-3 was downregulated (Fig. 3h). Thus, the expression and nuclear translocation of β-catenin cannot be regulated by Bcl-3, although both proteins have some functional characteristics in common. These results suggest that Wnt3a increased the Bcl-3 protein level and its nuclear translocation.

### Bcl-3 is required for β-catenin/TCF-mediated transcription and the expression of Wnt/β-catenin target genes

**Fig. 4:**
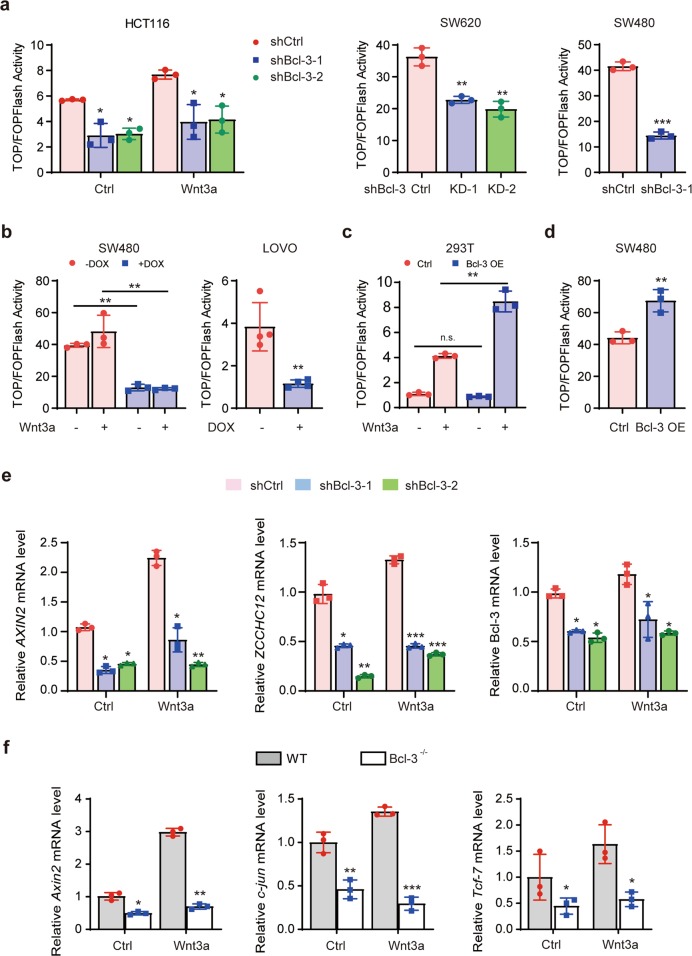
Bcl-3 is required for β-catenin/TCF-4-mediated transcription and the expression of Wnt/β-catenin target genes. **a** HCT116 and SW620 cells with two different shRNA sequences and SW480 cells with the shBcl-3 KD-1 sequence were cotransfected with TOP/Flash or FOP/Flash reporter and Renilla luciferase normalization control. After Wnt3a treatment for 24 h or not, total cell lysate was collected to measure the firefly and Renilla luciferase activities. **b** SW480 and LOVO cells with a doxycycline (Dox, 1 μg/ml)-inducible Tet-on KD system were used. **c**, **d** Cells were cotransfected with Bcl-3-Flag or control expression plasmid with TOP/Flash or FOP/Flash reporter, and Renilla luciferase normalization control. After Wnt3a treatment for 24 h or not, total cell lysate was collected to measure the firefly and Renilla luciferase activities. Values are the means ± SD for each cohort (*n* = 3). *p < 0.05 and **p < 0.01 by two-tailed Student’s *t*-test. **e** *AXIN2*, *ZCCHC12*, and *Bcl-3* mRNA expression levels were detected in Bcl-3 KD HCT116 cells with or without Wnt3a treatment for 24 h. **f** *Axin2*, c-jun, and *Tcf-7* mRNA expression levels were detected in wild-type and Bcl-3 knockout MEFs with or without Wnt3a treatment for 24 h. The results are shown as the means ± SD. *p < 0.05, **p < 0.01, and ***p < 0.001 by two-tailed Student’s *t*-test

Wnt activation-induced accumulation and nuclear translocation of β-catenin result in associations with members of the TCF/LEF family of DNA-bound transcription factors on TCF-binding elements to promote Wnt target gene expression.^35,36^ We used a TOP/Flash reporter to determine whether Bcl-3 affects β-catenin–TCF-4/LEF transcriptional activity. Interestingly, Bcl-3 KD strongly inhibited TOP/Flash reporter expression in the HCT116 cell line with or without Wnt3a treatment (Fig. 4a). The same result was confirmed in other CRC cell lines, SW620 and SW480, which have high levels of endogenous β-catenin (Fig. 4a). We also confirmed this result with the Dox-induced Tet-on shRNA system in SW480 and LOVO cells (Fig. 4b). Moreover, overexpression of Bcl-3 alone significantly activated the TOP/Flash reporter in SW480 cells (Fig. 4d). In the HEK293T cell line, the activation of the TOP/Flash reporter could only be induced after Wnt3a treatment when Bcl-3 was overexpressed (Fig. 4c); a possible explanation may be that HEK293T cells have barely detectable endogenous β-catenin activation in the absence of Wnt3a stimulation, which suggests that Bcl-3-mediated β-catenin–TCF-4 transcriptional activity should be initiated by Wnt activation.

Next, we examined the expression of several Wnt/β-catenin target genes in Bcl-3-silenced cell lines. The mRNA levels of *AXIN2* and *ZCCHC12* were decreased in HCT116 cells with Bcl-3 KD (Fig. 4e). Moreover, the expression of *Axin2, c-jun, and Tcf-7* was downregulated, and could not be induced after Wnt activation in Bcl-3 knockout MEFs (Fig. 4f). Together, these results indicate that Bcl-3 is required for β-catenin/TCF-mediated transcription and the expression of Wnt/β-catenin target genes.

### Bcl-3 interacts with β-catenin and is required for β-catenin recruitment to Wnt target gene promoters

**Fig. 5:**
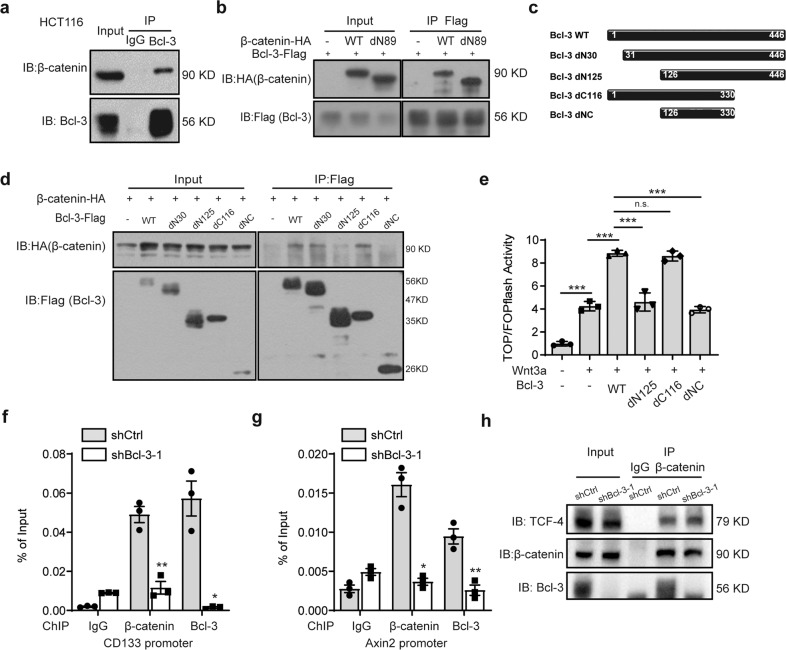
Bcl-3 interacts with β-catenin and is required for β-catenin recruitment to Wnt target gene promoters. **a** HCT116 cell lysates were prepared, and anti-Bcl-3 antibody was used in IP followed by immunoblot using the indicated antibodies. **b** Co-IP analyses of Bcl-3-Flag and β-catenin truncated expression vectors (HA-tagged β-catenin (WT) or N-terminal 89 amino acid deletion mutant of β-catenin) in 293 T cells and cell lysates with anti-Flag antibodies. **c** Representation of the wild-type Bcl-3 protein and Bcl-3 mutants. **d** Co-IP analyses of β-catenin-HA and Bcl-3-truncated expression vectors (WT: 1–446 amino acids; dN30: 31–446 amino acids; dN125: 126–446 amino acids; dC116: 1–330 amino acids, and dNC: 126–330 amino acids) in HEK293T cells, and cell lysates were immunoprecipitated with anti-Flag antibodies. **e** 293T cells were cotransfected with the indicated plasmids and TOP/Flash or FOP/Flash reporter plasmid, treated with Wnt3a for 24 h, and total cell lysates were collected to measure the firefly and Renilla luciferase activities. Values are means ± SD for each cohort (*n* = 3). ***p < 0.001 by two-tailed Student’s *t*-test. **f**, **g** ChIP assays on the promoter regions of the *CD133* and *AXIN2* genes were performed in control and Bcl-3-silenced HCT116 cells. **h** Co-IP analyses of the β-catenin and TCF-4 interaction in control and Bcl-3-silenced HCT116 cells

The IF analysis above showed that endogenous Bcl-3 and β-catenin were colocalized in both the cytoplasm and nucleus, regardless of Wnt3a stimulation (Fig. 3g). Then, we confirmed the interaction by immunoprecipitation (IP) analysis. In the HCT116 cell line, co-IP assays demonstrated that endogenous Bcl-3 bound to endogenous β-catenin (Fig. 5a). In addition, we cotransfected vectors encoding Flag-tagged Bcl-3, HA-tagged β-catenin (WT), or an N-terminal deletion mutant of β-catenin (dN89, transcriptionally active form of β-catenin lacking the N-terminal 89 amino acids^37^) into HEK293T cells, followed by IP of the cell lysates with an antibody against the HA tag. Immunoblot assays revealed that both WT and dN89 β-catenin could bind to Bcl-3 (Fig. 5b). Further mapping analysis revealed that an N-terminal deletion mutant of Bcl-3 (dN125, a mutant form of Bcl-3 lacking the N-terminal 125 amino acids) could not interact with β-catenin, while dN30 (a mutant form of Bcl-3 lacking the N-terminal 30 amino acids) Bcl-3 could still be found in the β-catenin complex (Fig. 5d). To ascertain whether Bcl-3 mutants affect the transcriptional activity of Wnt/β-catenin signaling, Bcl-3 full-length and mutant constructs were cotransfected with TOP/FOP Flash luciferase reporter vectors into 293T cells. Consistent with the mapping assay (Fig. 5d), overexpression of full-length Bcl-3 and dC116 (a mutant form of Bcl-3 lacking the C-terminal 116 amino acids) mutants strongly activated TOP/Flash reporter activity, whereas dN125 and dNC did not (Fig. 5e). Thus, these results indicate that Bcl-3 interacts with β-catenin and that the capability of Bcl-3 to promote Wnt/β-catenin transcriptional activity correlates with the binding of Bcl-3 and β-catenin. The chromatin immunoprecipitation (ChIP) assay was then performed in HCT116 cells, and we found that both Bcl-3 and β-catenin were recruited to the endogenous promoters of the CD133 and Axin2 genes (Fig. 5f, g). The association of β-catenin with gene promoters decreased upon Bcl-3 depletion (Fig. 5f, g). However, the co-IP analysis results indicated that Bcl-3 depletion did not influence the interaction of β-catenin and TCF-4 (Fig. 5h), which suggested that the target factor influenced by Bcl-3 might be β-catenin. Thus, these results show that Bcl-3 can form a complex with β-catenin to activate Wnt target gene expression by binding with gene promoters.

### Bcl-3 promotes Wnt signaling by regulating the K49 acetylation of β-catenin

**Fig. 6:**
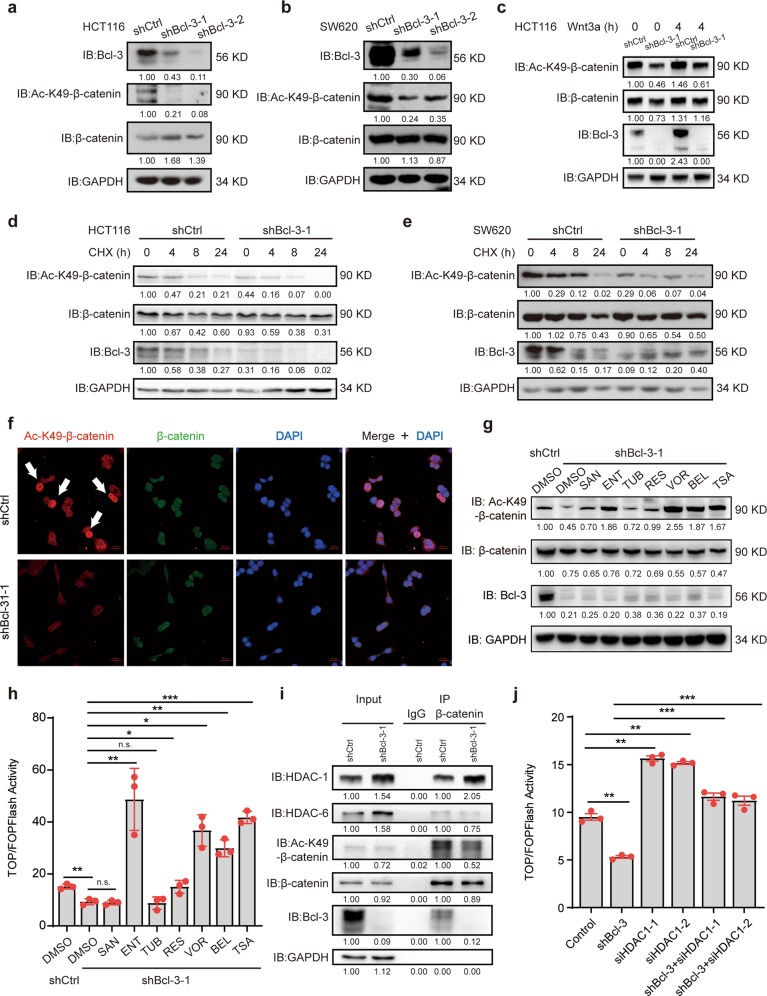
Bcl-3 promotes Wnt signaling by regulating the K49 acetylation of β-catenin. **a**, **b** Immunoblot analyses of Bcl-3, Ac-K49-β-catenin, and β-catenin in control and Bcl-3-silenced HCT116 cells **a** and SW620 cells **b**. **c** Immunoblot analyses of Bcl-3, Ac-K49-β-catenin, and β-catenin in control and Bcl-3-silenced HCT116 cells treated with Wnt3a for 4 h (100 ng/ml). **d**, **e** Immunoblot analyses of Bcl-3, Ac-K49-β-catenin, and β-catenin in control, and Bcl-3-silenced HCT116 cells and SW620 cells treated with CHX for the indicated time points. **f** Immunofluorescence staining of Ac-K49-β-catenin (red) and β-catenin (green) in control, and Bcl-3-silenced HCT116 cells. The nuclei were stained using DAPI (blue). **g** Immunoblot analyses of Bcl-3, Ac-K49-β-catenin, and β-catenin in control, and Bcl-3-silenced HCT116 cells treated with or without different HDAC inhibitors for 24 h; (SAN, santacruzamate A, 1 μM; ENT, entinostat, 5 μM; TUB, tubacin, 5 μM; RES, resminostat, 1 μM; VOR, vorinostat, 10 μM; BEL, belinostat, 1 μM; and TSA, 1 μM). **h** Control and Bcl-3-silenced HCT116 cells were cotransfected with TOP/Flash or FOP/Flash reporter and Renilla luciferase normalization control. After HDAC inhibitor treatment for 24 h, total cell lysate was collected to measure the firefly and Renilla luciferase activities. Values are means ± SD for each cohort (*n* = 3). **i** Lysates from control and Bcl-3-silenced HCT116 cells were prepared, and anti-β-catenin antibody was used in IP followed by immunoblot using the indicated antibodies. The results were quantified by ImageJ software. The relative protein levels in the input group were normalized to GAPDH levels. The relative protein levels in the IP group were normalized to β-catenin levels. **j** HCT116 cells were cotransfected with the indicated siRNA and TOP/Flash or FOP/Flash reporter plasmid, and then total cell lysates were collected to measure the firefly and Renilla luciferase activities. Values are means ± SD for each cohort (*n* = 3). *p < 0.05, **p < 0.01, and ***p < 0.001 by two-tailed Student’s *t*-test

As Bcl-3 did not affect the expression of β-catenin, we diverted our attention to the epigenetic modification of β-catenin. Since β-catenin can be acetylated at K49 by CREB-binding protein (CBP) and Ac-K49 β-catenin is required for activation of Wnt target genes,^38–40^ we detected the Ac-K49 β-catenin level when Bcl-3 was silenced. Notably, Bcl-3 depletion significantly decreased Ac-K49 β-catenin in both HCT116 and SW620 cells (Fig. 6a, b). The same results were observed in HCT116 cells treated with Wnt3a (Fig. 6c). When de novo protein synthesis was inhibited by CHX, the degradation of Ac-K49 β-catenin was remarkably faster in Bcl-3-silenced cells than in control cells (Fig. 6d, e). Similarly, the KD of Bcl-3 substantially decreased the level of Ac-K49 β-catenin, as determined by IF analysis (Fig. 6f). Moreover, CBP overexpression in Bcl-3 KD cells significantly increased the Wnt/β-catenin transcriptional activity (Supplementary Fig. 4a).

The small molecule ICG-001 inhibits the Wnt/β-catenin signaling pathway by first disrupting the interaction between CBP and β-catenin, and then decreasing the Ac-K49 β-catenin level.^41^ As shown in Supplementary Fig. 4b, ICG-001 treatment resulted in dramatically lower reporter activity, regardless of Bcl-3 overexpression (Supplementary Fig. 4b). Then, q-RT-PCR analysis of the *CD133* and *SOX2* genes indicated the same effect of ICG-001 on endogenous target gene expression (Supplementary Fig. 4c). The number of spheres in ICG-001-treated HCT116 cells was found to be >2-fold less than the number of spheres formed in DMSO-treated cells (Supplementary Fig. 4d).

Next, we examined how Bcl-3 regulates the expression of Ac-K49 β-catenin. First, we blocked ubiquitin-mediated proteasome activity by treating cells with MG-132 and a lysosome-mediated degradation system with chloroquine, followed by detecting Ac-K49 β-catenin levels. Surprisingly, the protein level of Ac-K49 β-catenin increased after MG-132 treatment of control cells but barely increased in Bcl-3-silenced cells. Moreover, Ac-K49 β-catenin was not changed in control cells or Bcl-3-silenced cells after chloroquine stimulation (Supplementary Fig. 4e).

We have shown that the inhibitor of Ac-K49 β-catenin ICG-001 could mimic the effect of Bcl-3 KD on the Wnt/β-catenin signaling pathway. Then, we considered the deacetylation modification of β-catenin. We used two inhibitors of histone deacetylase, tubastatin A (TSA, which inhibits HDACs) and niacinamide (NAM, which specifically inhibits SITRs), and analyzed the protein level of Ac-K49 β-catenin in Bcl-3 KD and control cells. As shown in Supplementary Fig. 4f, both NAM and TSA induced Ac-K49 β-catenin levels in control cells, and only TSA rescued the Ac-K49 β-catenin levels when Bcl-3 was knocked down. Then, we verified the effect of other HDAC inhibitors on Ac-K49 β-catenin levels in Bcl-3-depleted cells. Vorinostat and belinostat are two inhibitors targeting HDACs; resminostat inhibits HDAC-1, -3, and -6; entinostat inhibits HDAC-1 and -3; santacruzamate A inhibits HDAC-2; and tubacin is an inhibitor of HDAC-6. We treated the indicated cells with or without these inhibitors for 24 h and detected the expression of related proteins. The western blot assay indicated that an inhibitor of HDAC-1 and -3 (entinostat), an inhibitor of HDAC-1, -3, and -6 (resminostat), and three inhibitors of HDACs (vorinostat, belinostat, and TSA) could rescue the expression of Ac-K49 β-catenin when Bcl-3 was knocked down (Fig. 6g). Additionally, treatment with these five inhibitors significantly enhanced Wnt/β-catenin transcriptional activity in Bcl-3-silenced cells (Fig. 6h). The mRNA level of CD133 decreased in Bcl-3 KD cells, and it was upregulated after treatment with these five inhibitors (Supplementary Fig. 4g). These results indicated that HDACs, especially HDAC-1, may be involved in the process of β-catenin acetylation at Lys49 regulated by Bcl-3.

It has been reported that Bcl-3 forms a complex with HDAC-1, -3, and -6 to control the expression of a subset of genes.^34^ We hypothesized that Bcl-3 alters the interaction between β-catenin and HDACs. Co-IP assays showed that β-catenin bound to endogenous Bcl-3, HDAC-1, HDAC-6 (Fig. 6i), and HDAC-1 but not HDAC-6, which increased in the β-catenin complex in Bcl-3-silenced cells (Fig. 6i). Our results showed that both HDAC-1 and HDAC-6 protein levels increased when Bcl-3 was silenced. Moreover, the interaction of HDAC-1 and β-catenin was enhanced in Bcl-3 KD cells, but the binding of HDAC-6 and β-catenin did not change. These results indicated that in addition to inhibiting the expression of HDAC-1, Bcl-3 could also inhibit the binding of HDAC-1 to β-catenin, thus increasing the acetylation level of β-catenin.

Furthermore, the transcriptional activity of β-catenin was partially regulated by Bcl-3 via HDAC-1 (Fig. 6j). These results demonstrated that Bcl-3 regulated Ac-K49 β-catenin levels in a deacetylase-dependent manner whereby Bcl-3 segregated HDAC-1 and β-catenin to maintain a relatively high level of Ac-K49 β-catenin to promote Wnt/β-catenin activity.

### Bcl-3 is overexpressed in CRC tissues and correlates with CRC patient survival

**Fig. 7:**
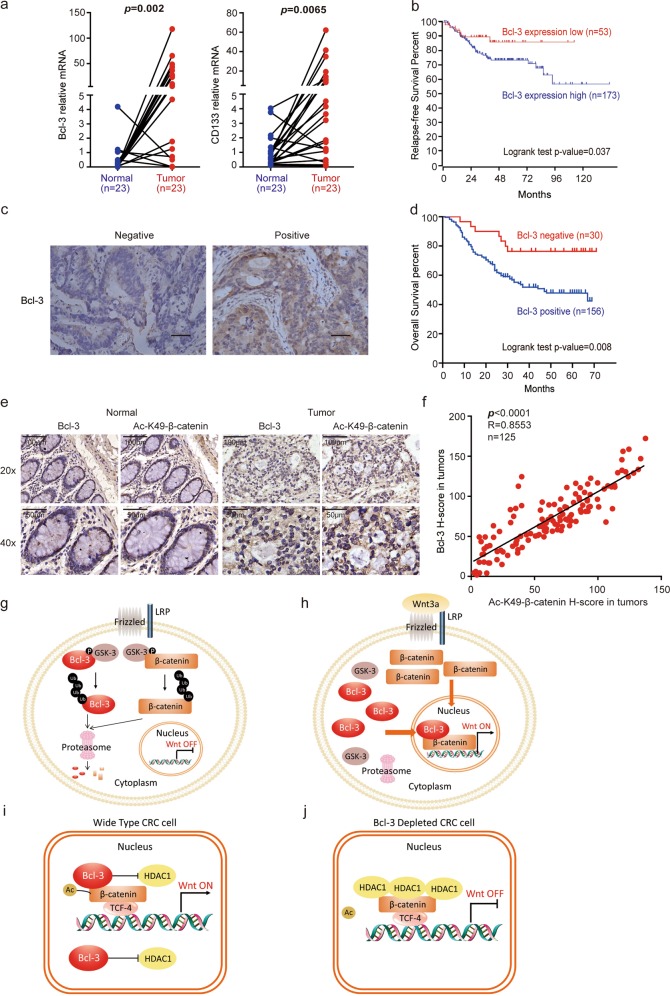
Bcl-3 is overexpressed in CRC tissues and correlates with CRC patient survival. **a** *Bcl-3* and *CD133* mRNA levels in 23 matched tumors and paired normal biopsies. *p* = 0.0009 for Bcl-3 and *p* = 0.0044 for *CD133* by paired *t*-test. **b** Kaplan–Meier graph shows the survival analyses of patients with colorectal cancer based on Bcl-3 expression using R2: Genomics Analysis and Visualization Platform. **c** Representative images of positive and negative Bcl-3 staining in CRC patients. **d** Kaplan–Meier graph shows the overall survival rate in CRC patients based on Bcl-3 staining using IHC assay. **e** Representative images of Bcl-3 and Ac-K49-β-catenin expression based on IHC of serial sections of tissue microarrays. **f** The expression of Bcl-3 and Ac-K49-β-catenin in CRC tissue microarrays was analyzed by Spearman rank correlation coefficient analysis; *R* = 0.8646; *p* < 0.001; *n* = 125. **g**, **h** Cartoon illustration of the mechanisms by which Wnt3a increases Bcl-3 protein levels. **i**, **j** Cartoon illustration of the mechanisms by which Bcl-3 regulates the Wnt/β-catenin signaling pathway

We then assessed *Bcl-3* mRNA expression based on the ONCOMINE Colorectal Dataset (https://www.oncomine.org/resource/main.html)^25^ and found that Bcl-3 mRNA in adenocarcinoma tissues was much higher than that in normal colorectal tissues (Supplementary Fig. 5). Subsequently, both *Bcl-3* and *CD133* mRNA levels were found to be significantly increased in human colorectal tumors compared with their paired normal biopsies (Fig. 7a).

To further assess the clinical relevance of Bcl-3 expression and CRC, we conducted a Kaplan–Meier survival analysis based on clinical data from GSE14333 through the bioinformatics website R2: Genomics Analysis and Visualization Platform (http://r2.amc.nl). Patients with higher levels of Bcl-3 mRNA (*n* = 173) showed significantly lower relapse-free survival than patients whose tumors expressed lower levels of Bcl-3 (*n* = 53; Fig. 7b). Then, 186 CRC patients (TMA-3) were categorized into positive and negative groups based on the protein expression of Bcl-3, and representative images are shown in Fig. 7c. The Kaplan–Meier survival rate supported the observation that Bcl-3 expression was significantly associated with poor survival in CRC patients (Fig. 7d).

We also examined the correlation between Bcl-3 and Ac-K49-β-catenin expression in human CRC. IHC analysis was performed for Bcl-3 and Ac-K49-β-catenin in serial sections of colon tumor microarrays, comprising 125 tumor specimens and 14 normal biopsies (the clinical information is summarized in Supplementary Table 1). Immunochemistry analyses revealed significantly higher Bcl-3 and Ac-K49-β-catenin protein levels in colorectal tumors than in normal colons (Fig. 7e). Consistent with our previous observation, a significantly positive correlation between Bcl-3 and Ac-K49-β-catenin was found in the tumor specimens (Fig. 7f). Taken together, these data indicate Bcl-3 as a potential biomarker for CRC, and the prognosis of patients with high Bcl-3 expression was worse than that of patients with low Bcl-3 expression.

## Discussion

Constitutive activation of Wnt signaling is closely associated with CRC progression and stem cell-like properties in CRC; therefore, understanding the biological basis for the observed Wnt signaling overactivation is of great value for future development of novel therapeutic strategies. Canonical Wnt/β-catenin signaling has been implicated in the regulation of CRC proliferation, maintenance of cellular stemness and chemoresistance. As Bcl-3 is overexpressed in many human cancers and regulates stemness of colorectal CSCs, we considered a direct connection between Bcl-3 and the Wnt/β-catenin signaling pathways. In this study, we demonstrated that Bcl-3 is required for Wnt/β-catenin transcriptional activity in CRC cells, which is achieved by maintaining the level of Ac-K49 β-catenin. In summary, without Wnt3a stimulation, both Bcl-3 and β-catenin were maintained at relatively low levels due to the phosphorylation of GSK-3 and degradation of the ubiquitin-proteasome pathway (Fig. 7g). Under Wnt3a treatment, both proteins were released from GSK-3 and the degradation complex. Next, accumulated Bcl-3 and β-catenin translocated into the nucleus to form the Bcl-3–β-catenin complex on the promoter of Wnt target genes (Fig. 7h). In wild-type CRC cell nuclei, Bcl-3 and β-catenin formed a transcriptional complex with TCF-4 on Wnt target gene promoters, and β-catenin was acetylated by CBP and p300 (Fig. 7i). When Bcl-3 was silenced, HDAC-1 was overexpressed, and more HDAC-1 bound with β-catenin to significantly decrease β-catenin acetylation levels, which resulted in suppressed gene expression (Fig. 7j).

Recently, another study addressed some of the same issues as those presented here.^42^ These two independent pieces of work arrived at essentially the same conclusions, further validating the results of both studies. However, our work demonstrated in detail that Bcl-3 promotes the colorectal CSC phenotype and chemoresistance by enhancing Wnt/β-catenin signaling. We further revealed the critical role of the acetylation of β-catenin at lysine 49 in Wnt/β-catenin signaling, which was modulated by Bcl-3.

Aside from the phosphorylation of GSK-3 at S394/398, other kinases, including Akt, Erk2, and IKK, participate in Bcl-3 phosphorylation and regulate its transcriptional activity.^43^ The phosphorylation of Bcl-3 at Ser33 through Akt could promote Bcl-3 stabilization and nuclear localization.^43^ Moreover, the deubiquitinase enzyme CYLD removes the K63-linked ubiquitin chain on Bcl-3 to prevent Bcl-3 nuclear accumulation.^43^ These results indicate areas for further exploration of the mechanism by which Wnt induces Bcl-3 nuclear translocation, and whether these kinases and phosphorylation sites on Bcl-3 are required for Wnt3a-induced Bcl-3 nuclear translocation also need further verification.

In the process of oligodendrocyte differentiation, HDAC-1 competes with β-catenin for TCF7L2 interaction to negatively regulate Wnt target gene expression.^44^ However, in breast and ovarian cancer, HDAC-1 and HDAC-7 are overexpressed in CSCs and are necessary to maintain CSCs. In our work, HDAC-1 increased in Bcl-3-silenced cells, and increased levels of HDAC-1 bound to β-catenin were found in Bcl-3-depleted cells. In mESCs, Bcl-3 depletion decreased the binding of acetylated histone 3 on the *Oct4* promoter.^20^ Unfortunately, we found that the level of acetylated H3 did not change in Bcl-3 KD cells (data not shown). Whether Bcl-3 influences the binding of acetylated H3 to the Wnt target gene promoter remains to be determined.

Because of the crucial role of Wnt signaling in CRC progression and colorectal CSC maintenance, inhibition of Wnt/β-catenin signaling by targeting Bcl-3 may offer a therapeutic approach in CRC treatment.

## Supplementary information

Supplementary material

## Data Availability

All data and materials are available to the researchers once published.
