# Supplementary material for: Bcl-3 promotes Wnt signaling by maintaining the acetylation of β-catenin at lysine 49 in colorectal cancer
Source: Signal Transduct Target Ther. 2020 May 1;5:52. doi: 10.1038/s41392-020-0138-6 (PMC7193563; doi:10.1038/s41392-020-0138-6)
Supplement: Supplementary file 1 — Supplementary material [file 41392_2020_138_MOESM1_ESM.docx]

Supplementary Materials for

**Bcl-3 promotes Wnt signaling through sustaining the acetylation of β-catenin at lysine 49 in colorectal cancer**

Xi Chen^1*^, Chen Wang^1, 3*^, Yuhang Jiang^1, 2^, Qi Wang^1^, Yu Tao^1,^ ^2^, Haohao Zhang^1,^ ^2^, Yongxu Zhao^1^, Yiming Hu^1, 2^, Cuifeng Li^1, 2^, Deji Ye^1^, Dandan Liu^1^, Wenxia Jiang^1^, Eugene Y Chin^1^, Sheng Chen^4^, Yongzhong Liu^5^, Mingliang Wang^6#^, Sanhong Liu^1, 3#^ and Xiaoren Zhang^1, 2#^

^1^CAS Key Laboratory of Tissue Microenvironment and Tumor, Shanghai Institute of Nutrition and Health, Shanghai Institutes for Biological Sciences, University of Chinese Academy of Sciences, Chinese Academy of Sciences, Shanghai, 200025, China;

^2^Affiliated Cancer Hospital & Institute of Guangzhou Medical University, Guangzhou Municipal and Guangdong Provincial Key Laboratory of Protein Modification and Degradation, State Key Laboratory of Respiratory Disease, Guangzhou, 510000, China;

^3^Shanghai Institute of Advanced Immunochemical Studies, ShanghaiTech University, Shanghai 201210, China;

^4^Renji Hospital, Shanghai Jiao Tong University School of Medicine, Shanghai, 200025, China;

^5^State Key Laboratory of Oncogenes and Related Genes, Shanghai Cancer Institute, Renji Hospital, Shanghai Jiao Tong University School of Medicine, 200032, Shanghai, China;

^6^Department of General Surgery, Ruijin Hospital, Shanghai Jiao Tong University School of Medicine, Shanghai, 200025, China.

* These authors contributed equally

Correspondence to:

Xiaoren Zhang^1, 2^ (e-mail: xrzhang@sibs.ac.cn)

Sanhong Liu^1, 3^ (e-mail: liush@shanghaitech.edu.cn)

Mingliang Wang^5^ (e-mail: wml_2902@163.com)

**This PDF file includes:**

Materials and Methods

Figures. S1 to S5

Tables S1

Materials and Methods

**Cell lines and reagents**

The human colorectal cancer cell lines HCT116, SW480, SW620 and LOVO, HEK-293T cells were purchased from the ATCC. Cell lines were cultured in Dulbecco’s Modified Eagle’s Medium (DMEM, HyClone, Logan, UT, USA) contained 10% FBS (SH300700, HyClone, Logan, UT), 1% penicillin and streptomycin (#15140122, Gibco). Reagents used were as followed: Recombinant Wnt3a (#5036/WN, R&D Systems), oxaliplatin (HY-17371, MedChemExpress), 5-fluorouracil (5-FU, S1209, Selleck), cycloheximide (C7698, Sigma), MG-132 (C2211, Sigma), ICG-001 (S2662, Selleck), Nicotinamide (NAM, S1899, Selleck), Trichostatin A (TSA, S1045, Selleck), Santacruzamate A (S7595, Selleck), Entinostat (S1053, Selleck), Tubacin (S2239, Selleck), Resminostat (S2693, Selleck), Vorinostat (S1047, Selleck), Belinostat (S1085, Selleck), Chloroquine diphosphate (HY-17589, MedChemExpress).

**Plasmids construction and Lentiviral transduction**

Two different shRNA sequences against Bcl-3 and a non-silencing shRNA sequence was cloned into plvx-shRNA plasmid. The third Bcl-3 shRNA sequences was cloned into the pTRIPZ inducible lentiviral shRNA plasmid (Dharmacon, RHS4750, Huntsville). The sequences are noted below.

shBcl-3-1:

F-5’-GATCCGAAGGACCCTTACACTCTTTTCAAGAGAAAGAGTGTAAGGGTCCTTCTTTTTTG-3’;

R-5’-AATTCAAAAAAGAAGGACCCTTACACTCTTTCTCTTGAAAAGAGTGTAAGGGTCCTTCCG-3’;

shBcl-3-2:

F-5’-GATCCGCGACATCTACAACAACCTATTCAAGAGATAGGTTGTTGTAGATGTCGTTTTTTG-3’;

R-5’-AATTCAAAAAACGACATCTACAACAACCTATCTCTTGAATAGGTTGTTGTAGATGTCGCG-3’;

shBcl-3-3:

F-5’-GAGCTCGAACCAACCTAAAGAAAACATTATCACTTCGGTGTCTACATAATGTTTTCTTTAGGTTGG

TTTCTTAAG-3’;

R-5’-CTTAAGAAACCAACCTAAAGAAAACATTATGTAGACACCGAAGTGATAATGTTTTCTTTAGGTTG

GTTCG AGCTC-3’.

The lentivirus was produced using shRNA plasmids, psPAX2 and pMD2G and stable cell lines were generated as described previously[^15^](#_ENREF_15). Flag-tagged Bcl-3 and Flag-tagged Bcl-3 mutant plasmids were cloned into pcDNA3.1. HA-tagged β-catenin and HA-tagged dN-β-catenin were kind gifts from Dr. Weijun Pan. TOP/Flash and FOP/Flash reporter plasmids were kind gifts from Dr. Lin Li.

**Immunoprecipitation and immunoblotting**

Immunoprecipitation and immunoblotting assays were performed as described previously[^15^](#_ENREF_15). The following primary antibodies were included: Bcl-3 (sc-185, Santa Cruz Biotechnology), Bcl-3 (#23959-1-AP, Proteintech Group), phospho-Bcl-3 (sc-33883, Santa Cruz Biotechnology), β-catenin (c2206, Sigma), β-catenin (sc-7963, Santa Cruz Biotechnology), K49 acetyl-β-catenin (#9030, Cell Signaling Technology), GAPDH (KC-5G4, KANGCHEN), Flag (F3165, Sigma), HA (sc-805, Santa Cruz Biotechnology), Lamin A (sc-7292, Santa Cruz Biotechnology), ubiquitin-K48 (#05-1307, Millipore), ubiquitin-K63 (#05-1308, Millipore), CD133 (#64326, Cell Signaling Technology), SOX2 (#3579, Cell Signaling Technology), HDAC-1 (P30116S, Abmart), HDAC-6 (#12834-1-AP, Proteintech Group) and TCF-4 (#05-511, Millipore). The secondary antibodies used were as followed: donkey anti-Goat IgG (KC-GT-035, KANGCHEN), goat anti-Mouse IgG (KC-MM-035, KANGCHEN) and goat anti- Rabbit IgG (KC-RB-035). Reagents used for immunoprecipitation were as followed: protein A-sepharose (GE17-1279-01, Sigma), Rabbit IgG (sc-2027, Santa Cruz Biotechnology), Mouse IgG (sc- 2025, Santa Cruz Biotechnology).

For ubiquitination assay, to rule out the possibility that these ubiquitination bands were from Bcl-3 associated protein rather than Bcl-3 itself, the protein mixture was firstly heated in the presence of 1% SDS and 5mM DTT to disassociate the protein-protein interactions, before being subjected to immunoprecipitation[^45^](#_ENREF_45).

**RNA extraction and quantitative real-time PCR**

Total RNA was isolated using TRIzol Reagent (15596-018; Invitrogen) following protocols of the manufacturer. RNA was reverse transcribed to cDNA by the PrimeScript^TM^ RT reagent Kit (RR047A, TaKaRa). Quantitative real-time PCR for target genes were performed using SYBR Green (RR420A, TaKaRa). Fold changes were calculated using the equation RQ=2^−ΔΔCt^. Each sample was analyzed in triplicate.

**Chromatin immunoprecipitation (ChIP)**

10 million cells were fixed for 10 minutes with 1% formaldehyde, then terminated the fix procedure with the addition of 0.125 M of glycine and washed with PBS. The chromatin immunoprecipitation was performed according to the manufacturer instruction of EZ-ChIP kit (#17-371, Millipore). The antibodies used for immunoprecipitation were Bcl-3 (sc-185, Santa Cruz Biotechnology), β-catenin (c2206, Sigma) and control IgG. The genomic DNA get from the ChIP were analyzed with q-RT-PCR with specific primers.

**Immunofluorescence assays**

Cells were plated and grown on coverslips, then washed with PBS and fixed with 4% paraformaldehyde. Cells were blocked with PBS containing 1% FBS and 0.3% Triton-X-100 for 30 min. Immunostaining was performed as described previously[^15^](#_ENREF_15). The primary antibodies used were as followed: Bcl-3 (#23959-1-AP, Proteintech Group), β-catenin (c2206, Sigma), K49 acetyl-β-catenin (#9030, Cell Signaling Technology). The secondary antibodies were coupled to AlexaFluor^®^488 or AlexaFluor^®^680. Fluorescent cells were observed with Zeiss laser-scanning confocal microscope (LSM Meta 510).

**FACS analysis**

FACS analyses were performed with a MoFlo Astrios Flow Cytometer (Beckman). CD133^+^CD44^+^ double positive cells were analyzed with CD133-PE (#130-090-853, Miltenyi Biotec) and CD44-APC (#560890, BD Transduction Laboratories) antibodies. Apoptosis was analyzed with Annexin V-FITC (#556420, BD Transduction Laboratories) and 7AAD (#559925, BD Transduction Laboratories).

**Luciferase reporter assay**

CRC cells were cultured in 24-well dishes and transfected with 250 ng of TOP/FOP Flash reporter plasmids and 50 ng of Renilla plasmids as an internal control using Fugene (#04709705001, Roche). 24 h post transfection, cells were treated with 100 ng/ml Wnt3a for 24 hours and cell lysates were collected at 48 hours after transfection. Then the luciferase activities were detected with the Dual-Luciferase Reporter System (#07311, Promega). The TOP/FOP ratio was used to measure the Wnt/β-catenin transcriptional activity.

**Immunohistochemistry**

Immunohistochemistry assays were performed on the paraffin-embedded CRC tissue as previously described[^15^](#_ENREF_15). The primary antibodies included Bcl-3 (#23959-1-AP, Proteintech Group), Ac-K49-β-catenin (#9030, Cell Signaling Technology). The degree of immunostaining of indicated proteins was scored by H-SCORE, which was calculated using the formula: H-SCORE = (percentage of cells of weak intensity x 1) + (percentage of cells of moderate intensity x 2) + (percentage of cells of strong intensity x 3). The H-SCORE would between 0-300, 300 stands for 100% of cells with strong intensity.

**Sphere formation assay**

1 x 10^4^ CRC cells were seeded in ultra-low adherent 24-well plates (Corning) for 7 days. Cells were cultured in serum-free medium containing DMEM/F12 (#15-090-CM, Cellgro), 2% B27 Supplement (#12587010, Invitrogen), 20 ng/ml EGF (#315-09, Peprotech) and 10 ng/ml FGF (#345-FG-025, R&D).

**Tumor xenografts *in vivo***

Animal experiments were approved by the institutional biomedical research ethics committee of Shanghai Institutes for Biological Sciences (Shanghai, China). Three doses of the indicated cells (5 x 10^5^, 5 x 10^4^, 5 x 10^3^) were injected subcutaneously in 6-8-week BALB/c nude mice (male, n=5 per group) respectively. Tumor size were detected every week using a digital caliper and tumor volumes were calculated with the equation: V= (length [mm] x width^2^ [mm^2^])/2. The mice were sacrificed at 4 weeks post injection.

**Statistical analyses**

Statistical analyses were performed using GraphPad Prism 6.0 (GraphPad Software, Inc.). Unless otherwise indicated, all experiments were repeated three times and all data were presented as means ± SD and comparisons between two groups were performed by two-tailed Student’s t-test. For more than two groups, we used one-way ANOVA or two-way ANOVA with Tukey’s multiple comparisons test. CRC patient survival probability was analyzed by the Kaplan-Meier method and the significance was assessed using the log-rank test. IHC results were analyzed using a Chi-square test. The expression of Bcl-3 and Ac-K49-β-catenin in CRC patient samples was analyzed for correlation by Spearman rank correlation coefficient analysis. For all statistical tests, p<0.05 was considered statistically significant.

Figure. S1.


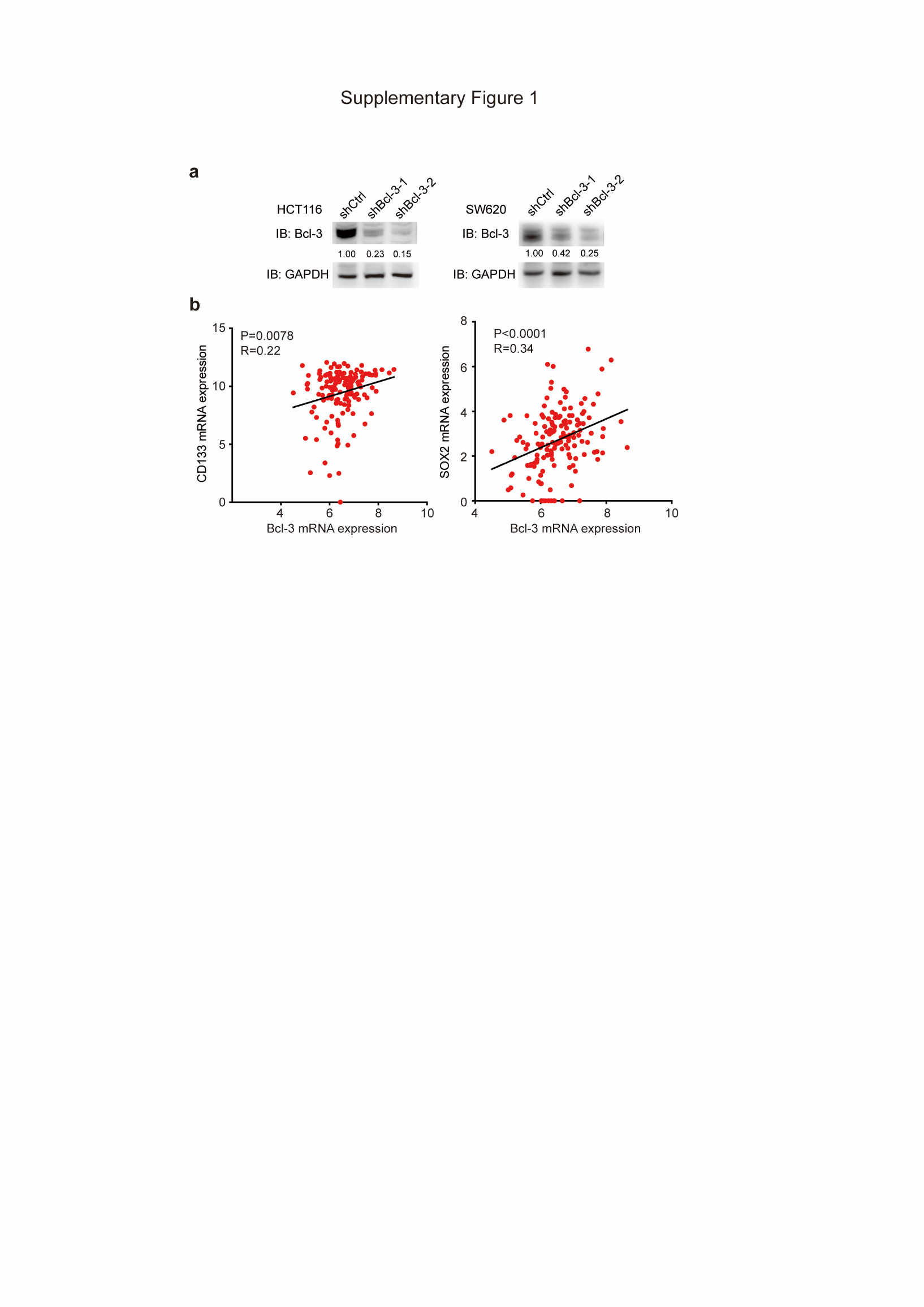


**Figure S1. Bcl-3 maintains stem cell like properties in CRC cells *in vitro.***

1. Immunoblot analyses of Bcl-3 in control and Bcl-3 silenced HCT116 cells and SW620 cells.
2. Scatter plots of *Bcl-3* mRNA expressions versus mRNA expression levels of *CD133* and *SOX2* in colorectal adenocarcinoma (n=148) from Genomics Analysis and Visualization Platform dataset were plotted.

Figure. S2.


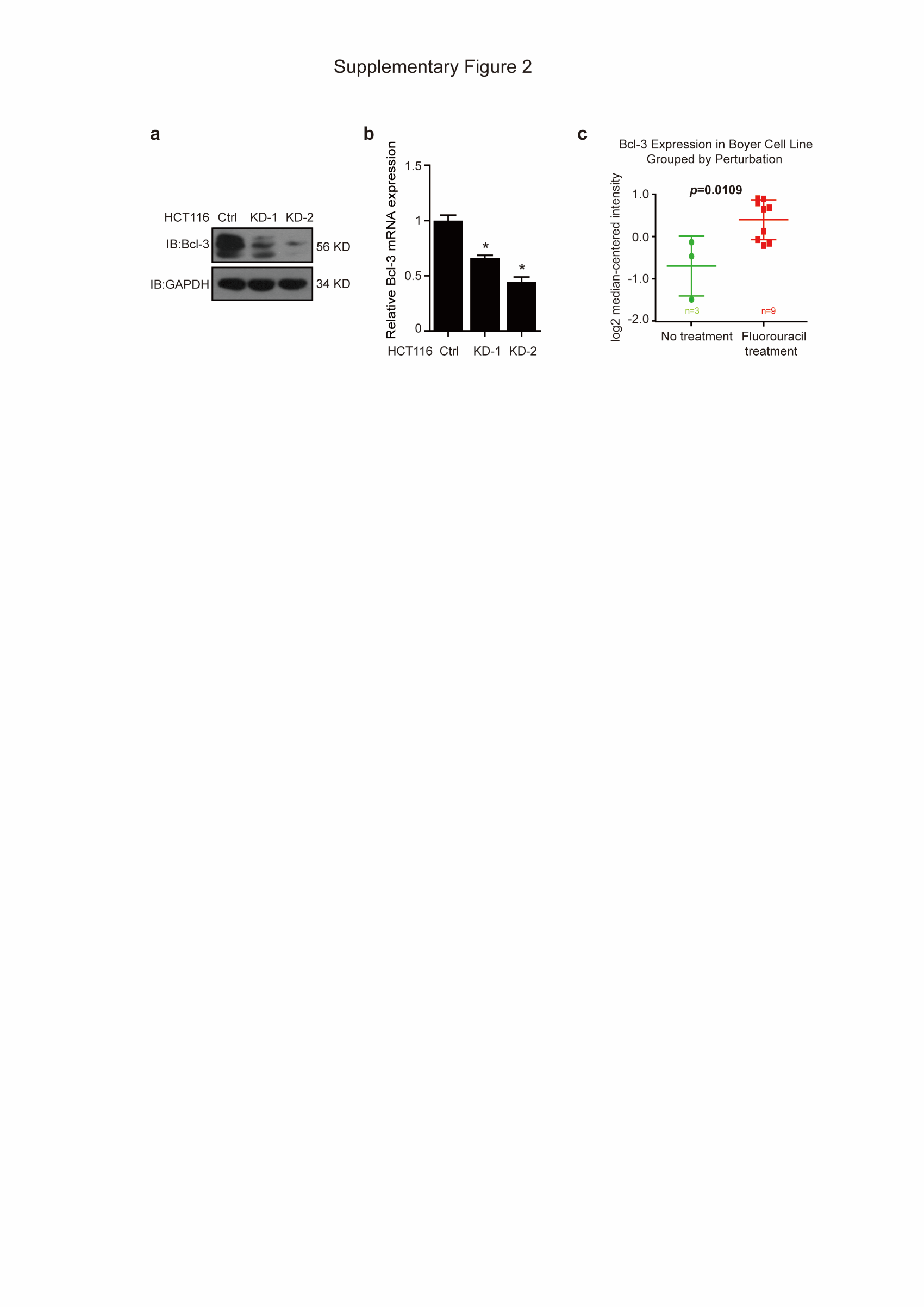


**Figure S2.** **Bcl-3 enhances the tumorigenicity and chemoresistance of CRC *in vivo.***

1. Immunoblots for Bcl-3 in control and Bcl-3 knocked down HCT116 cells.
2. q-RT-PCR analysis for Bcl-3 in control and Bcl-3 knocked down HCT116 cells.
3. mRNA expression (log2) of Bcl-3 from ONCOMINE database in Fluorouracil treated cells.

Figure. S3.


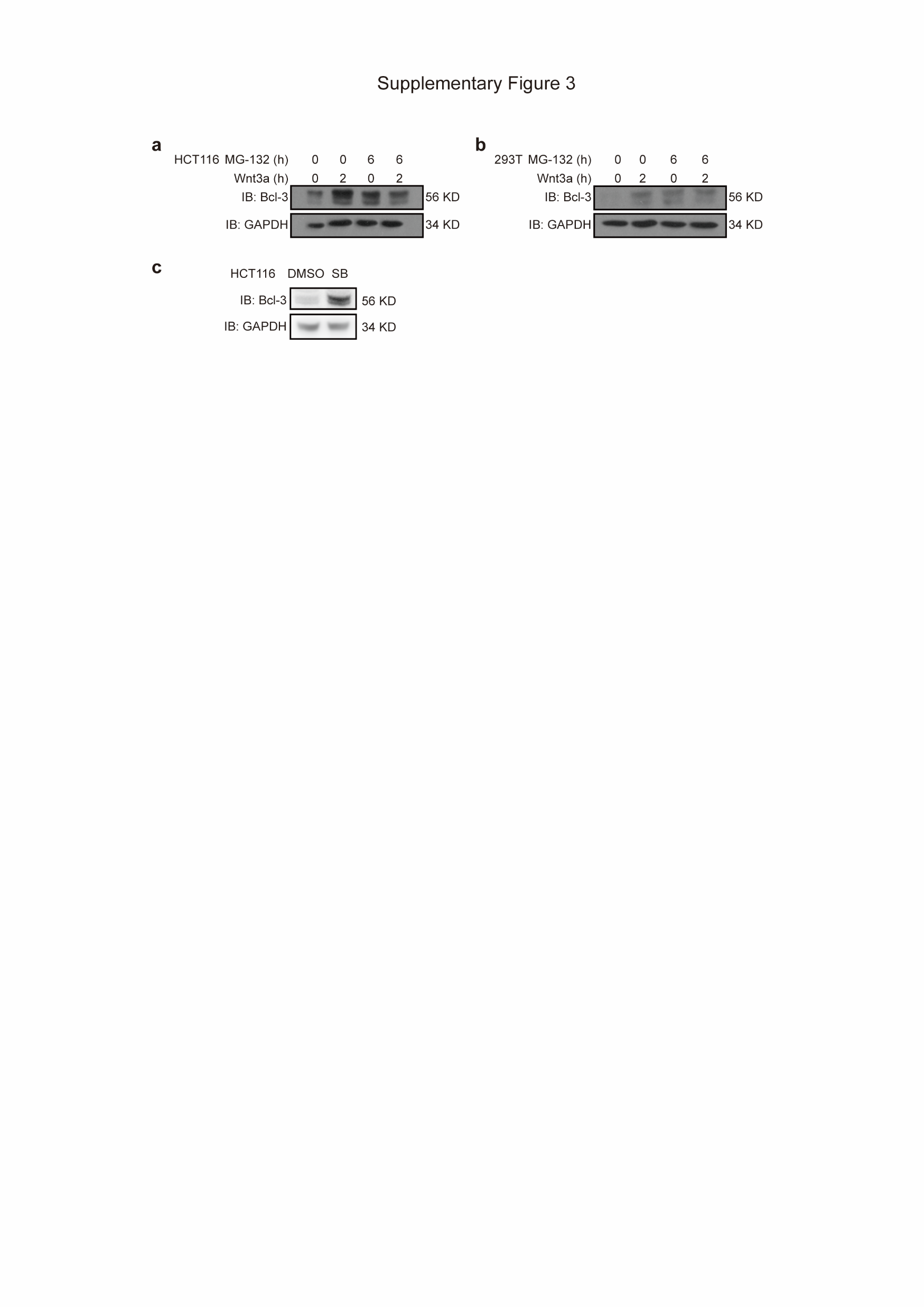


**Figure S3. Wnt3a induced increase in Bcl-3 protein rely on GSK-3 kinase activity.**

a-b. Immunoblots for Bcl-3 in HCT116 (**a**) and 293T (**b**) cells treated with Wnt3a and MG-132 for indicated time points.

c. Immunoblots for Bcl-3 in HCT116 cells treated with SB216763 (5 μM) for 24 hours.

Figure. S4.


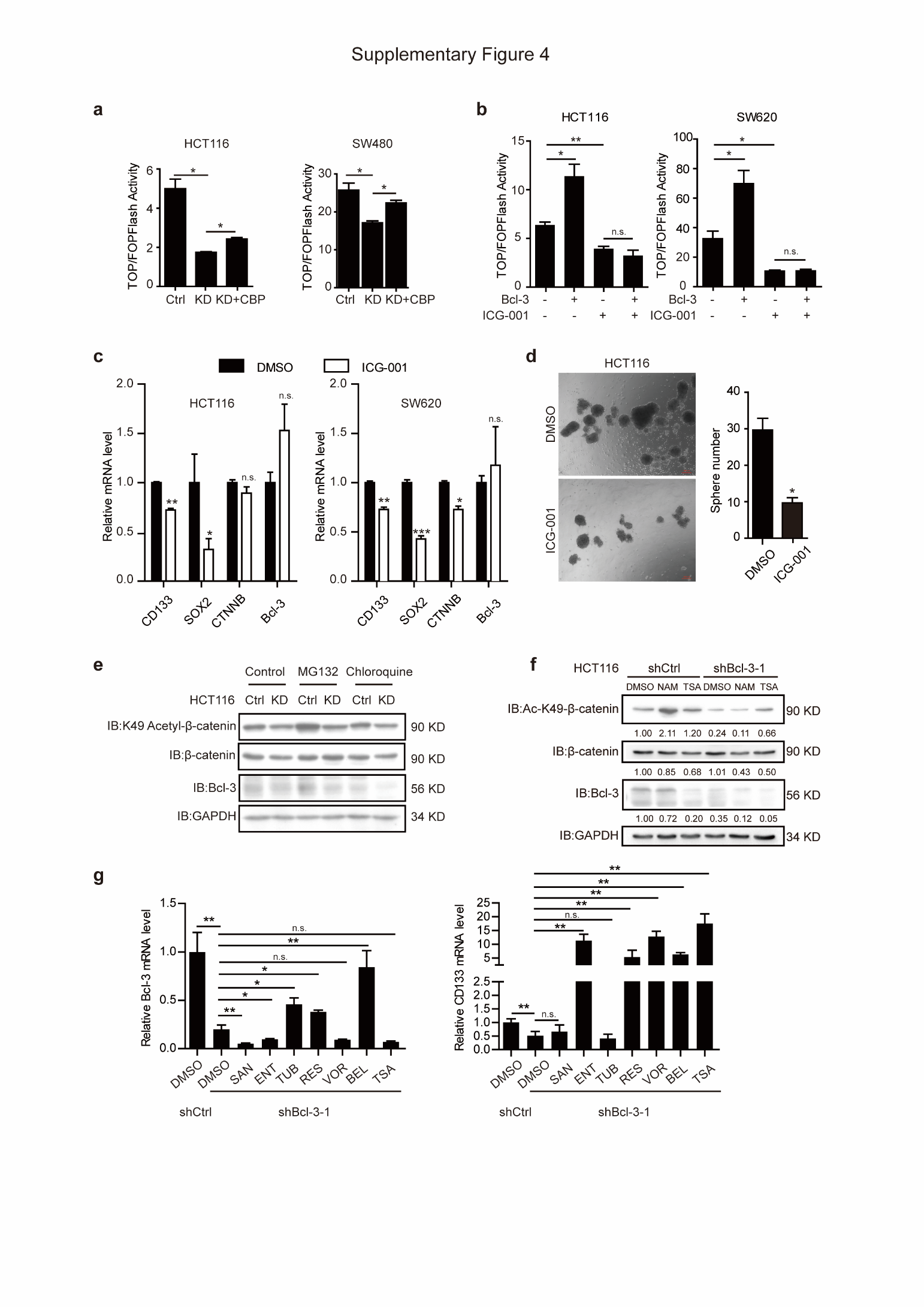


**Figure S4. Bcl-3 promotes Wnt signaling through regulating K49-acetylation of β-catenin.**

1. CBP overexpression activating the TOP/Flash reporter luciferase activity in Bcl-3 silenced

HCT116 and SW480 cells. Cells were cotransfected with CBP-HA or control expression plasmid with TOP/Flash or FOP/Flash reporter and Renila luciferase normalization control. 48 hours post transfection total cell lysate were collected to measure the firefly luciferase and Renila activities. Values are means ± SD for each cohort (n=3). **p* < 0.05 by two-tailed Student’s *t*-test.

b. ICG-001 inhibits the TOP/Flash reporter luciferase activity in HCT116 and SW620 cells. Cells were cotransfected with Bcl-3-Flag or control expression plasmid with TOP/Flash or FOP/Flash reporter and Renila luciferase normalization control. After ICG-001 (50 μM) stimulated for 24 hours total cell lysate were collected to measure the firefly luciferase and Renila activities. Values are means ± SD for each cohort (n=3). **p* < 0.05, ***p* < 0.01 by two-tailed Student’s *t*-test.

**c.** *CD133*, *SOX2, CTNNB1* and *Bcl-3* mRNA expression were detected in ICG-001 treated HCT116 and SW620 cells. The results are shown as means ± SD. **p* < 0.05, ***p*<0.01 and ****p* <0.001 by two-tailed Student’s *t*-test.

**d**. ICG-001 treatment causes a reduced sphere-forming capacity in HCT116 cells.

**e**. Immunoblot analyses of Bcl-3, Ac-K49-β-catenin and β-catenin in control and Bcl-3 silenced HCT116 cells treated with MG-132 (5 μM for 24 fours) or Chloroquine (1 mM for 24 hours).

**f**. Immunoblot analyses of Bcl-3, Ac-K49-β-catenin and β-catenin in control and Bcl-3 silenced HCT116 cells treated with NAM (10 μM for 24 fours) or TSA (1 μM for 24 hours).

**g**. *Bcl-3* and *CD133* mRNA expression were detected in shCtrl and shBcl-3-1 cells treated with or without different HDAC inhibitors for 24 hours. (SAN, Santacruzamate A, 1 μM; ENT, Entinostat, 5 μM; TUB, Tubacin, 5 μM; R, RESesminostat, 1 μM; VOR, Vorinostat, 10 μM; BEL, Belinostat, 1 μM; TSA, 1 μM).

Figure. S5.


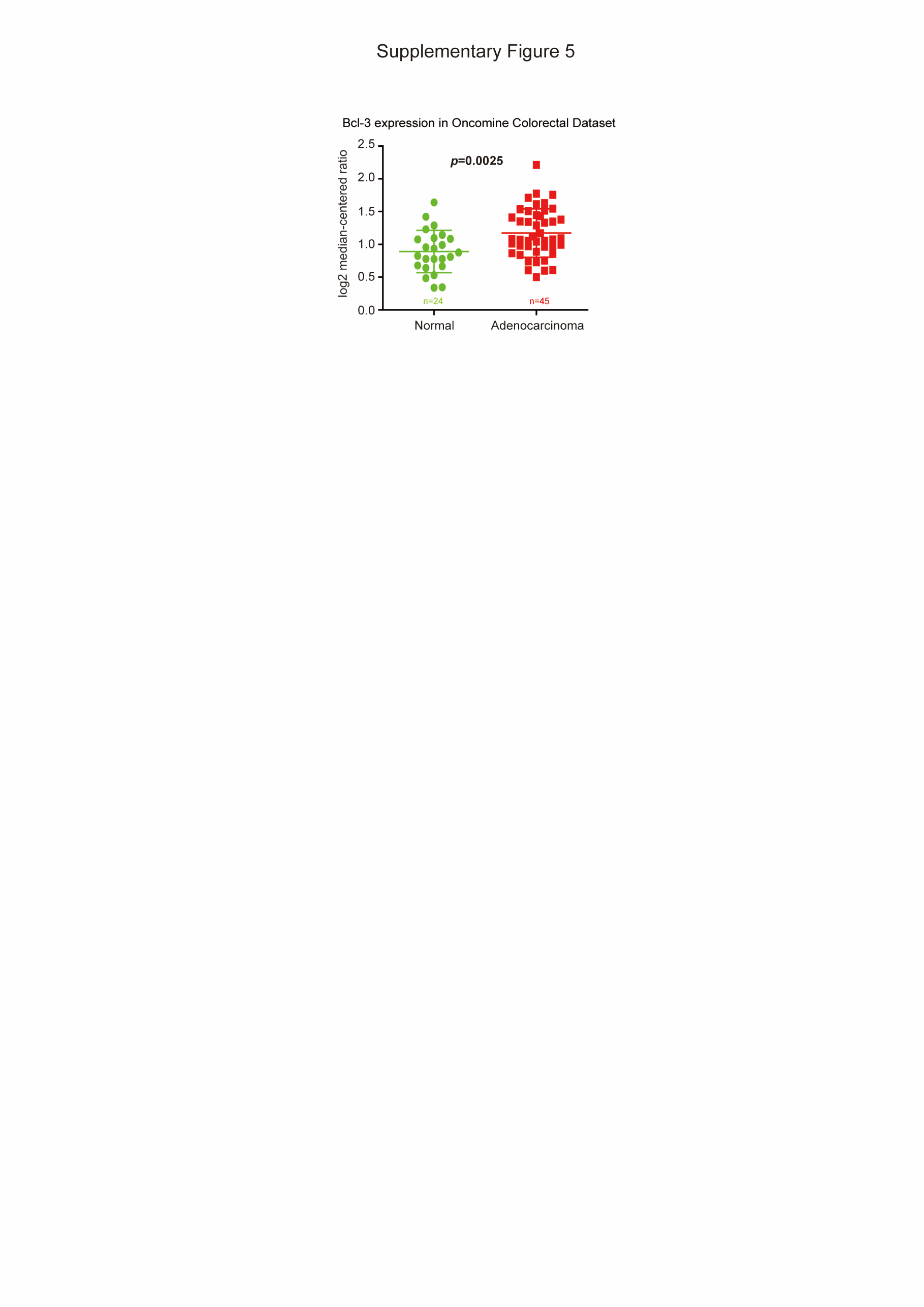


**Figure S5.** **Bcl-3 is overexpressed in CRC tissues and correlates with CRC patient survival.**

The mRNA expression (log_2_) of Bcl-3 from ONCOMINE database in colorectal cancer versus normal tissues.

Table S1. Clinical information of CRC patients.

| **No.** | **Age** | **Sex** | **Organ(Anatomic Site)** | **Pathology diagnosis** | TNM | Grade | Stage | Type |
| --- | --- | --- | --- | --- | --- | --- | --- | --- |
| 1 | 42 | F | Colon | Adenocarcinoma | T4N0M0 | 1 | IIB | Malignant |
| 2 | 65 | M | Colon | Mucinous adenocarcinoma | T3N0M0 | 1 | IIA | Malignant |
| 3 | 70 | M | Colon | Adenocarcinoma | T3N0M0 | 1 | IIA | Malignant |
| 4 | 50 | F | Colon | Adenocarcinoma | T4N2M0 | 1 | IIIC | Malignant |
| 5 | 53 | F | Colon | Adenocarcinoma | T4N0M0 | 1 | IIB | Malignant |
| 6 | 34 | M | Colon | Adenocarcinoma | T4N0M0 | 1 | IIB | Malignant |
| 7 | 72 | M | Colon | Adenocarcinoma | T2N0M0 | 1 | I | Malignant |
| 8 | 51 | M | Colon | Adenocarcinoma | T4N2M0 | 1 | IIIC | Malignant |
| 9 | 27 | M | Colon | Adenocarcinoma | T4N0M0 | 1 | IIB | Malignant |
| 10 | 70 | M | Colon | Adenocarcinoma | T3N1M0 | 1 | IIIB | Malignant |
| 11 | 60 | F | Colon | Adenocarcinoma | T3N0M0 | - | IIA | Malignant |
| 12 | 53 | F | Colon | Adenocarcinoma | T3N0M0 | 1 | IIA | Malignant |
| 13 | 71 | M | Colon | Adenocarcinoma | T3N0M0 | 1 | IIA | Malignant |
| 14 | 60 | M | Colon | Adenocarcinoma | T3N0M0 | 1 | IIA | Malignant |
| 15 | 30 | F | Colon | Adenocarcinoma | T3N2M0 | 1 | IIIC | Malignant |
| 16 | 55 | M | Colon | Adenocarcinoma | T2N0M0 | 1 | I | Malignant |
| 17 | 49 | F | Colon | Adenocarcinoma | T4N1M0 | 1 | IIIB | Malignant |
| 18 | 75 | M | Colon | Adenocarcinoma | T4N0M0 | 1 | IIB | Malignant |
| 19 | 44 | M | Colon | Adenocarcinoma | T4N0M0 | - | IIB | Malignant |
| 20 | 82 | M | Colon | Adenocarcinoma | T4N0M0 | 1 | IIB | Malignant |
| 21 | 86 | M | Colon | Adenocarcinoma | T4N1M0 | 1 | IIIB | Malignant |
| 22 | 68 | F | Colon | Adenocarcinoma | T3N1M0 | 1 | IIIB | Malignant |
| 23 | 65 | F | Colon | Mucinous adenocarcinoma | T3N0M0 | 1 | IIA | Malignant |
| 24 | 61 | M | Colon | Mucinous adenocarcinoma | T3N0M0 | 1 | IIA | Malignant |
| 25 | 59 | F | Colon | Mucinous adenocarcinoma | T3N1M0 | 1 | IIIB | Malignant |
| 26 | 32 | M | Colon | Mucinous adenocarcinoma | T3N0M0 | 1 | IIA | Malignant |
| 27 | 58 | M | Colon | Mucinous adenocarcinoma | T4N1M1 | 1 | IV | Malignant |
| 28 | 55 | M | Colon | Mucinous adenocarcinoma | T3N2M0 | 2 | IIIC | Malignant |
| 29 | 63 | F | Colon | Adenocarcinoma | T4N0M0 | 1 | IIB | Malignant |
| 30 | 40 | F | Colon | Mucinous adenocarcinoma | T3N1M0 | 1 | IIIB | Malignant |
| 31 | 71 | M | Colon | Adenocarcinoma | T4N2M0 | 2 | IIIC | Malignant |
| 32 | 61 | M | Colon | Adenocarcinoma | T4N0M0 | 2 | IIB | Malignant |
| 33 | 86 | F | Colon | Adenocarcinoma | T4N0M0 | 2 | IIB | Malignant |
| 34 | 64 | M | Colon | Mucinous adenocarcinoma | T4N0M0 | 1 | IIB | Malignant |
| 35 | 52 | F | Colon | Adenocarcinoma | T3N0M0 | 2 | IIA | Malignant |
| 36 | 46 | M | Colon | Adenocarcinoma | T4N0M0 | 2 | IIB | Malignant |
| 37 | 70 | F | Colon | Adenocarcinoma | T4N0M0 | 1 | IIB | Malignant |
| 38 | 70 | M | Colon | Adenocarcinoma | T3N0M0 | 2 | IIA | Malignant |
| 39 | 75 | M | Colon | Adenocarcinoma | T4N0M0 | 2 | IIB | Malignant |
| 40 | 51 | F | Colon | Adenocarcinoma (sparse) | T4N0M0 | - | IIB | Malignant |
| 41 | 60 | M | Colon | Adenocarcinoma | T4N0M0 | 2 | IIB | Malignant |
| 42 | 41 | F | Colon | Adenocarcinoma (sparse) | T4N0M0 | - | IIB | Malignant |
| 43 | 60 | F | Colon | Adenocarcinoma | T4N0M0 | 2 | IIB | Malignant |
| 44 | 48 | F | Colon | Adenocarcinoma | T3N0M0 | 2 | IIA | Malignant |
| 45 | 66 | M | Colon | Mucinous adenocarcinoma | T3N0M0 | 2 | IIA | Malignant |
| 46 | 73 | M | Colon | Adenocarcinoma | T4N1M0 | 2 | IIIB | Malignant |
| 47 | 75 | M | Colon | Adenocarcinoma | T3N0M0 | 2 | IIA | Malignant |
| 48 | 37 | M | Colon | Adenocarcinoma | T4N1M0 | - | IIIB | Malignant |
| 49 | 44 | F | Colon | Adenocarcinoma | T3N0M0 | 1 | IIA | Malignant |
| 50 | 35 | M | Colon | Adenocarcinoma | T4N1M0 | - | IIIB | Malignant |
| 51 | 53 | F | Colon | Adenocarcinoma | T3N0M0 | 1 | IIA | Malignant |
| 52 | 70 | M | Colon | Adenocarcinoma | T3N0M0 | 1 | IIA | Malignant |
| 53 | 40 | F | Colon | Adenocarcinoma | T4N0M0 | 1 | IIB | Malignant |
| 54 | 53 | M | Colon | Mucinous adenocarcinoma | T3N0M0 | 3 | IIA | Malignant |
| 55 | 28 | M | Colon | Mucinous adenocarcinoma | T4N0M0 | 3 | IIB | Malignant |
| 56 | 71 | F | Colon | Adenocarcinoma | T3N0M0 | 2 | IIA | Malignant |
| 57 | 58 | M | Colon | Adenocarcinoma | T4N0M0 | 2 | IIB | Malignant |
| 58 | 74 | M | Colon | Adenocarcinoma | T3N0M0 | 2 | IIA | Malignant |
| 59 | 69 | M | Colon | Adenocarcinoma | T4N2M0 | 2 | IIIC | Malignant |
| 60 | 66 | M | Colon | Adenocarcinoma | T3N0M0 | 2 | IIA | Malignant |
| 61 | 71 | F | Colon | Mucinous adenocarcinoma | T3N0M0 | 2 | II | Malignant |
| 62 | 76 | M | Colon | Mucinous adenocarcinoma | T3N0M0 | 2 | IIA | Malignant |
| 63 | 76 | F | Colon | Adenocarcinoma | T2N0M0 | 1 | I | Malignant |
| 64 | 50 | F | Colon | Mucinous adenocarcinoma | T4N0M0 | 2 | IIB | Malignant |
| 65 | 60 | F | Colon | Mucinous adenocarcinoma | T3N1M0 | 2 | IIIB | Malignant |
| 66 | 49 | F | Colon | Mucinous adenocarcinoma | T4N1M1 | 3 | IV | Malignant |
| 67 | 77 | M | Colon | Adenocarcinoma with necrosis | T3N1M0 | 2 | IIIB | Malignant |
| 68 | 80 | F | Colon | Adenocarcinoma | T3N0M0 | 2 | IIA | Malignant |
| 69 | 70 | F | Colon | Adenocarcinoma | T3N2M0 | 2 | IIIC | Malignant |
| 70 | 30 | M | Colon | Adenocarcinoma | T2N1M0 | 2 | IIIA | Malignant |
| 71 | 43 | M | Colon | Adenocarcinoma | T3N1M0 | 2 | IIIB | Malignant |
| 72 | 50 | M | Colon | Adenocarcinoma | T2N1M1 | 2 | IV | Malignant |
| 73 | 41 | M | Colon | Adenocarcinoma | T4N2M0 | 2 | IIIC | Malignant |
| 74 | 66 | M | Colon | Adenocarcinoma with necrosis | T3N0M0 | - | IIA | Malignant |
| 75 | 79 | M | Colon | Adenocarcinoma | T3N0M0 | 1 | IIA | Malignant |
| 76 | 49 | M | Colon | Adenocarcinoma | T4N0M0 | 1 | IIB | Malignant |
| 77 | 56 | F | Colon | Adenocarcinoma | T3N0M0 | - | IIA | Malignant |
| 78 | 73 | F | Colon | Adenocarcinoma | T4N2M0 | 2 | IIIC | Malignant |
| 79 | 77 | M | Colon | Adenocarcinoma | T3N0M0 | 2 | IIA | Malignant |
| 80 | 50 | M | Colon | Adenocarcinoma | T2N0M0 | 1 | I | Malignant |
| 81 | 48 | M | Colon | Adenocarcinoma | T3N0M0 | 2 | IIA | Malignant |
| 82 | 44 | M | Colon | Adenocarcinoma | T4N0M0 | 2 | IIB | Malignant |
| 83 | 31 | M | Colon | Adenocarcinoma | T4N0M0 | 2 | IIB | Malignant |
| 84 | 82 | M | Colon | Adenocarcinoma | T3N0M0 | 2 | IIA | Malignant |
| 85 | 71 | M | Colon | Adenocarcinoma | T4N2M0 | 2 | IIIC | Malignant |
| 86 | 62 | M | Colon | Adenocarcinoma | T3N0M0 | 2 | IIA | Malignant |
| 87 | 47 | M | Colon | Adenocarcinoma | T4N0M0 | 2 | IIB | Malignant |
| 88 | 55 | M | Colon | Adenocarcinoma | T3N1M0 | 2 | IIIB | Malignant |
| 89 | 62 | M | Colon | Adenocarcinoma | T4N0M1 | 2 | IV | Malignant |
| 90 | 38 | M | Colon | Adenocarcinoma | T4N1M0 | 2 | IIIB | Malignant |
| 91 | 50 | M | Colon | Adenocarcinoma | T4N0M0 | 2 | II | Malignant |
| 92 | 70 | M | Colon | Mucinous adenocarcinoma | T4N0M0 | 2 | IIB | Malignant |
| 93 | 22 | F | Colon | Adenocarcinoma | T4N2M1 | 3 | IV | Malignant |
| 94 | 58 | M | Colon | Adenocarcinoma | T3N1M0 | 3 | IIIB | Malignant |
| 95 | 33 | M | Colon | Adenocarcinoma | T3N0M0 | 3 | IIA | Malignant |
| 96 | 72 | M | Colon | Adenocarcinoma | T4N0M0 | 3 | IIB | Malignant |
| 97 | 68 | M | Colon | Adenocarcinoma | T4N0M1 | 3 | IV | Malignant |
| 98 | 54 | M | Colon | Mucinous adenocarcinoma | T3N1M1 | 3 | IV | Malignant |
| 99 | 24 | M | Colon | Mucinous adenocarcinoma | T3N2M0 | 3 | IIIC | Malignant |
| 100 | 56 | M | Colon | Adenocarcinoma | T3N0M0 | 3 | IIA | Malignant |
| 101 | 45 | M | Colon | Mucinous adenocarcinoma | T3N0M0 | 3 | IIA | Malignant |
| 102 | 68 | M | Colon | Adenocarcinoma | T3N1M0 | 3 | III | Malignant |
| 103 | 36 | M | Colon | Adenocarcinoma | T4N0M0 | 3 | IIB | Malignant |
| 104 | 74 | F | Colon | Adenocarcinoma with necrosis | T4N1M0 | 3 | IIIB | Malignant |
| 105 | 60 | M | Colon | Adenocarcinoma with necrosis | T3N0M0 | 3 | IIA | Malignant |
| 106 | 53 | F | Colon | Adenocarcinoma | T4N0M0 | 3 | IIB | Malignant |
| 107 | 49 | M | Colon | Mucinous adenocarcinoma | T4N1M0 | 3 | IIIB | Malignant |
| 108 | 71 | M | Colon | Adenocarcinoma | T4N1M0 | 2 | IIIB | Malignant |
| 109 | 75 | M | Colon | Adenocarcinoma | T4N0M0 | 3 | IIB | Malignant |
| 110 | 31 | F | Colon | Adenocarcinoma (sparse) | T4N1M1 | - | IV | Malignant |
| 111 | 35 | M | Colon | Colon tissue | - | - | - | Normal |
| 112 | 25 | M | Colon | Colon tissue | - | - | - | Normal |
| 113 | 35 | M | Colon | Colon tissue | - | - | - | Normal |
| 114 | 30 | M | Colon | Colon tissue | - | - | - | Normal |
| 115 | 30 | M | Colon | Colon tissue | - | - | - | Normal |
| 116 | 40 | M | Colon | Colon tissue | - | - | - | Normal |
| 117 | 28 | M | Colon | Colon tissue | - | - | - | Normal |
| 118 | 45 | M | Colon | Colon tissue | - | - | - | Normal |
| 119 | 33 | M | Colon | Colon tissue | - | - | - | Normal |
| 120 | 30 | M | Colon | Colon tissue | - | - | - | Normal |
| 121 | 76 | F | Colon | Tubular-villous adenocarcinoma | T3N1M0 | 1 | III | malignant |
| 122 | 46 | M | Colon | Mucinous adenocarcinoma | T4N1M0 | 3 | III | malignant |
| 123 | 45 | F | Colon | Mucinous adenocarcinoma | T3N0M0 | 1 | IIA | malignant |
| 124 | 69 | F | Colon | Adenocarcinoma | T3N1M0 | 2 | III | malignant |
| 125 | 76 | F | Colon | Tubular-villous adenocarcinoma | T3N1M0 | 1 | III | malignant |
| 126 | 46 | M | Colon | Mucinous adenocarcinoma | T4N1M0 | 3 | III | malignant |
| 127 | 45 | F | Colon | Mucinous adenocarcinoma | T3N0M0 | 1 | IIA | malignant |
| 128 | 69 | F | Colon | Adenocarcinoma (fibrofatty tissue) | T3N1M0 | - | III | malignant |
| 129 | 73 | M | Colon | Tubular adenocarcinoma | T2N0M0 | 1--2 | I | malignant |
| 130 | 50 | F | Colon | Adenocarcinoma (fibrous tissue and blood vessel) | T3N1M0 | - | III | malignant |
| 131 | 66 | F | Colon | Tubular adenocarcinoma | T3N0M0 | 2 | IIA | malignant |
| 132 | 51 | M | Colon | Adenosquamous carcinoma | T3N0M0 | - | IIA | malignant |
| 133 | 73 | M | Colon | Tubular adenocarcinoma | T2N0M0 | 1--2 | I | malignant |
| 134 | 50 | F | Colon | Adenocarcinoma (sparse) | T3N1M0 | 2 | III | malignant |
| 135 | 66 | F | Colon | Tubular adenocarcinoma | T3N0M0 | 2 | IIA | malignant |
| 136 | 30 | M | Colon | Normal colon tissue | - | - | - | normal |
| 137 | 25 | M | Colon | Normal colon tissue | - | - | - | normal |
| 138 | 30 | M | Colon | Normal colon tissue | - | - | - | normal |
| 139 | 25 | M | Colon | Normal colon tissue | - | - | - | normal |
